# Fast and robust phase-shift estimation in two-dimensional structured illumination microscopy

**DOI:** 10.1371/journal.pone.0221254

**Published:** 2019-08-16

**Authors:** Jorge Sola-Pikabea, Arcadi Garcia-Rius, Genaro Saavedra, Jorge Garcia-Sucerquia, Manuel Martínez-Corral, Emilio Sánchez-Ortiga

**Affiliations:** 1 Department of Optics, 3D Imaging and Display Laboratory, Universitat of València, Valencia (Burjassot), Spain; 2 School of Physics, Universidad Nacional de Colombia, Sede Medellin, Medellin, Colombia; Friedrich-Schiller-Universitat Jena, GERMANY

## Abstract

A method of determining unknown phase-shifts between elementary images in two-dimensional Structured Illumination Microscopy (2D-SIM) is presented. The proposed method is based on the comparison of the peak intensity of spectral components. These components correspond to the inherent structured illumination spectral content and the residual component that appears from wrongly estimated phase-shifts. The estimation of the phase-shifts is carried out by finding the absolute maximum of a function defined as the normalized peak intensity difference in the Fourier domain. This task is performed by an optimization method providing a fast estimation of the phase-shift. The algorithm stability and robustness are tested for various levels of noise and contrasts of the structured illumination pattern. Furthermore, the proposed approach reduces the number of computations compared to other existing techniques. The method is supported by the theoretical calculations and validated by means of simulated and experimental results.

## Introduction

Breaking the diffraction limit [1–3] has been successfully achieved in modern microscopy by different methods over the last couple of decades. As a result, non-conventional techniques that push the resolution of optical microscopes beyond the classical limit have been proposed [4–11]. Amongst them, structured illumination (SI) has been shown as a powerful alternative that can double the resolution of wide-field microscopes. SI does not require for a focused beam in the illumination, which reduces photo-bleaching of the fluorophores and allows a larger field-of-view imaging [8–17] compared to scanning techniques. In SI the sample is illuminated by a structured pattern generated by a coherent superposition of planes waves. The illumination pattern interacts with a fluorescent sample, resulting in an incoherent field which corresponds to a modulated version of the object irradiance distribution. This modulation shifts high-spatial frequencies of the object spectrum into low-frequency components that pass through the transmission band of the wide-field optical transfer function (OTF) of the microscope. However, these components are mixed together with the wide-field spectrum in the band support of the OTF. Therefore, a phase-shifting technique must be applied to untangle the different spatial frequency components. Consequently, several images (named elementary images) with relative phase-shifts of the illumination pattern must be taken to obtain a single reconstruction with enhanced resolution. Once the spatial frequency components are isolated, an extended image spectrum can be obtained with a resolution higher than the limit imposed by the diffraction barrier. Nevertheless, the goodness of the final reconstruction is strictly related to the precision in the knowledge of the introduced phase-shifts. Errors in the determination of these values produce artifacts in the final reconstruction and an erroneous estimation of the object [18]. Several methods can be applied for determining the phase-shifts from the elementary images, even with no prior knowledge of the system. Shroff, Fienup, & Williams [19] proposed the measurement of the phase in the secondary spectral peaks of the elementary images to calculate the estimated phase-shifts. This method is robust and fast, but produces errors that can be up to 10% for small phase-shifts. In additions, the method accuracy significantly decreases for SI patterns of spatial frequency higher than 85% of the cut-off frequency of the OTF. This estimation error is especially self-defeating inasmuch as it limits the resolution improvement that can be obtained with SIM: doubling the spatial resolution implies the use of a spatial frequency of the structured pattern that matches the cut-off frequency of the wide-field OTF. To solve these drawbacks, Wicker, Mandula, Best, Fiolka, & Heintzmann [18] proposed a robust method which can estimate the phase-shifts when the peaks of the SI spectral components are not clearly visible or even fall out of the support region of the OTF. This approach consisted in minimizing the cross-correlation between the calculated components by iteratively changing the estimated phase-shift. A variant of the method that used a single-step was proposed by Wicker [20], but results could be less precise than utilizing an iterative process, especially in low-photon/high-noise conditions. In this paper we present a fast and robust method for the estimation of the phase-shifts between elementary images in 2D-SIM. We validated the method by simulated and experimental data. The algorithm robustness is tested under different conditions such as the contrast of the pattern, noise and expected number of photons collected by the optical system.

## Materials and methods

### Theory

Let us briefly review the theoretical background and principles of 2D-SIM. In this type of microscope, a thin fluorescent sample with a density distribution of fluorophores *O*(**x**) is illuminated by a structured irradiance pattern *S*(**x**), being **x** = (*x*, *y*) the transverse coordinates. Note that the sample is assumed to be two-dimensional and therefore the axial behavior is neglected. The resulting intensity is imaged by the microscope, producing the following intensity distribution at the image plane

$$\begin{array}{c} I\left(x\right)=[O(\frac{x}{M})\cdot S(\frac{x}{M})]⊗h\left(x\right)\;, \end{array}$$ (1)

being *M* the lateral magnification of the imaging system, and ⊗ the 2D-convolution product. Furthemore, the 2D-point spread function (2D-PSF) is given by $h\left(x\right)=|\tilde{p}{\left(x\right)/\lambda f|}^{2}$, where $\tilde{p}$ is the Fourier transform of the microscope aperture stop, λ is the emission wavelength and *f* is the focal lenght of the tube-lens.

Typically, the structured illumination pattern *S*(**x**) is a harmonic function which can be expressed as the square modulus of a coherent superposition of plane waves. In practice, there are two cases of interest: the superposition of two plane waves traveling with a symmetrical direction with respect to the optical axis (2D-SIM), and the same two plane waves plus a third one traveling along the optical axis (3D-SIM). In this manuscript we will focus our attention in the first case which yields to two-dimensional improvement of the optical resolution. Note that the extension of the presented method to 3D-SIM is not straightforward.

In the case of 2D-SIM, considering that the relative phase *ϕ* of the plane waves can be shifted, the illumination pattern can be described as

$$\begin{array}{c} S_{\phi }^{j}\left(x\right)=2(1+c\cos(2\pi MA^{j}\cdot x+\phi ))\;, \end{array}$$ (2)

being **A** the vector that defines the travelling direction of the plane waves and *c* the contrast of the resulting pattern, which can be affected by the coherence between the plane waves as well as their relative intensity. The index *j* refers to different directions of the pattern with respect to the optical axis that are required for obtaining an isotropic 2D-SIM. Note that the spatial frequency of the pattern, **A**, is defined in the image space and therefore the magnification of the system is included in Eq (2). Hence, the intensity distribution in the image plane can be written in the following form

$$\begin{array}{c} I_{\phi }^{j}\left(x\right)=[O(\frac{x}{M})\cdot S_{\phi }^{j}(\frac{x}{M})]⊗h\left(x\right)\;. \end{array}$$ (3)

The effect of the modulation in terms of the spatial frequency content of the image can be better understood in the spatial-frequency domain. Omitting irrelevant constant factors, the Fourier transform of the image intensity distribution is given by

$$\begin{array}{c} {\tilde{I}}_{\phi }^{j}\left(u\right)=[\tilde{O}(Mu)⊗{\tilde{S}}_{\phi }^{j}(Mu)]H\left(u\right)\;, \end{array}$$ (4)

where ∼ represents the 2D Fourier transform, **u** are the spatial frequency coordinates, and being *H*(**u**) the 2D optical transfer function (OTF) of the microscope. Inserting the Fourier transform of the illumination pattern in its analytical form and considering an ideal contrast of the pattern *c* = 1, Eq (4) can be written as follows

$$\begin{array}{c} {\tilde{I}}_{\phi }^{j}\left(u\right)=\sum_{m=-1}^{1}\frac{e^{-im\phi }}{|m|+1}C_{m}^{j}\left(u\right)\;, \end{array}$$ (5)

being

$$\begin{array}{c} C_{m}^{j}\left(u\right)=[\tilde{O}\left(Mu\right)⊗\delta (u+mA^{j})]H(u)\;. \end{array}$$ (6)

As it can be noted from Eq (6), apart from the wide-field spectrum of the object, *m* = 0, the modulation introduces high spatial frequency information of the object within the OTF support, namely, the SI components (*m* = −1, +1). This frequency shift is proportional to the pattern spatial frequency, **A**, which is chosen to be practically identical to the cut-off frequency of the OTF to optimize the resolution improvement. Although the components are mixed within the same spectral bandwidth, the dependence on the relative phase of the structured pattern in Eq (5) permits to separate them as solutions of a system of three linearly independent equations. A combination of three images with proper phase-shifts of the illumination pattern is required for this system. In a general case, the three phase-shifts between the elementary images are unknown. Thus, they can be written as *ϕ* = (*ϕ*_0_ − *α*, *ϕ*_0_, *ϕ*_0_ + *β*). Note that the way in which the phase-shifts are defined is arbitrary as it depends on the origin of the first phase-shift. For simplicity, we neglected the global phase (*ϕ*_0_ = 0), which does not affect the forthcoming mathematics. If the relative phase-shifts are known with high accuracy, the three components can be retrieved by solving the system of equations as a combination of the elementary images, $I_{-\alpha }^{j},I_{0}^{j},I_{+\beta }^{j}$, leading to

$$\begin{array}{c} R(\begin{array}{c} \begin{array}{cc} & C_{-1}^{j}\\ & C_{0}^{j}\\ & C_{+1}^{j} \end{array} \end{array})\left(u\right)=(\begin{array}{c} \begin{array}{cc} & {\tilde{I}}_{-\alpha }^{j}\\ & {\tilde{I}}_{0}^{j}\\ & {\tilde{I}}_{+\beta }^{j} \end{array} \end{array})\left(u\right)\;, \end{array}$$ (7)

being

$$\begin{array}{c} R=(\begin{array}{c} \begin{array}{cccc} & e^{i\alpha }/2\; & 1\; & e^{-i\alpha }/2\\ & 1/2 & 1\; & 1/2\\ & e^{-i\beta }/2 & 1\; & e^{i\beta }/2 \end{array} \end{array}) \end{array}$$ (8)

the matrix that solves the system.

Once the components of the spectrum are isolated, they must be repositioned and properly weighted to compensate for the shape of the OTF. The latter can be achieved by using a Wiener-like filter with either the measured or the estimated OTF of the system, namely $\hat{H}\left(u\right)$. In addition, an apodization filter, *H*_SI_(**u**), is multiplied by the resulting object spectrum. We chose an apodization filter based on the calculation of an OTF of size identical to the theoretical cut-off frequency of the 2D-SIM. Nonetheless, other choices can be made such as a triangular apodization function [15]. Note that this procedure is defined for one direction of the structured pattern given by the spatial frequency vector **A**^*j*^. The pattern is typically rotated three times to produce a quasi-isotropic enhancement of the resolution. Conventionally, three wave vectors, *j* = (1, 2, 3), are taken to produce relative rotations of the pattern equivalent to the angles −60°, 0°, 60°. These operations result in the estimated high-resolution object spectrum which can be expressed as

$$\begin{array}{c} {\tilde{O}}_{SI}^{j}\left(u\right)=\{\sum_{j=1}^{3}\sum_{m=-1}^{+1}\frac{C_{m}^{j}\left(u\right){\hat{H}}^{*}\left(u\right)}{{\left|\hat{H}\left(u\right)\right|}^{2}+w^{2}}⊗\delta (u-mA^{j})\}H_{SI}\left(u\right)\;, \end{array}$$ (9)

where *w* is the Wiener parameter. The intensity distribution of the object produced by SIM is calculated by simply computing the inverse Fourier transform of the Eq (9). Ideally, if the spatial frequency of the projected patterns is equal to the cut-off spatial frequency of the original OTF, the image intensity distribution obtained by SIM doubles the resolution with respect to the wide-field image.

### Normalized peak intensity difference maximization

In this section, we propose an alternative method of detecting the phase-shift between the elementary images in 2D-SIM. To this end, let us consider a 2D-SIM system which provides images that can be described in the terms stated in Section 2. Typically, the spectrum of an elementary image contains three measurable peaks as a result of the zero component of the object spectrum, shifted to three spectral positions determined by the SI spatial frequency, see Eq (5). The respective positions of the peaks for a given direction of the pattern *j* are placed in the coordinates provided by the vector $p_{m}^{j}=mA^{j}$, being *m* = (−1, 0, 1). If the peaks are measurable their spectral position can be detected with high accuracy.

Once the peak positions are known, elementary images with three relative phase-shifts *ϕ* = (−*α*, 0, *β*) are used as inputs for solving the system of equations. Since the values *α* and *β* are unknown, we solve the system for arbitrary values of the phase-shifts, *ϕ*′ = (−*α*′, 0, *β*′), namely the phase-shifts estimation. Then Eq (7) gives rise to an estimation of the solutions, that are the true solutions only if *α*′ = *α* and *β*′ = *β*. In such a case, the three components of the spectrum can be perfectly isolated from each other. Any other value of the estimated phase-shifts result in a combination of mixed components located at positions previously determined by the vector. In case of estimated phase-shifts deviating from the experimental values, the reconstruction algorithm leads to an improper unmixing of the components. Consequently, peaks at the zero frequency positions (i.e. shifted along the spatial frequency vector **A**) of all SIM orders appear in each unmixed SIM order. Hence, the presence of residual peaks indicates a mismatching between the experimental phase-shifts and the phase-shifts used in the reconstruction algorithm. Taking into account this reasoning, the intensities of the SI peak component and the residual peak for a given direction of the pattern, *j*, can be calculated by

$$\begin{array}{c} \begin{array}{c} \begin{array}{cc} & i_{peak}^{m}=|{\hat{C}}_{+1}(u=mA)|\;, \end{array} \end{array} \end{array}$$ (10)

where ${\hat{C}}_{+1}$ represents the estimated +1 SI component given by Eq (7), that is, the estimated solution of the system of equations. Note that even though a similar approach could be done with the wide-field component ${\hat{C}}_{0}$, we focus our attention on the high-resolution components. Since the solutions for ${\hat{C}}_{+1}$ and ${\hat{C}}_{-1}$ are completely symmetrical, the method is only applied to one of the components. We define the normalized peak intensity difference as

$$\begin{array}{c} \epsilon (\alpha^{\prime },\beta^{\prime })=\frac{i_{peak}^{+1}(\alpha^{\prime },\beta^{\prime })-i_{peak}^{-1}(\alpha^{\prime },\beta^{\prime })}{i_{peak}^{+1}(\alpha^{\prime },\beta^{\prime })+i_{peak}^{-1}(\alpha^{\prime },\beta^{\prime })}\;. \end{array}$$ (11)

In order to write the normalized peak intensity difference in an analytical form, let us consider the solution of the system of equations described in Eq (7). As we assumed the real phase-shifts *α* and *β* to be unknown, we introduce the estimated phase-shifts, *α*′ and *β*′, in order to solve the system. For instance, the system can be solved by using the Cramer’s rule. To this end, we calculated the determinant of the matrix in Eq (8), which results in *δ*(*α*′, *β*′) = *i*[sin *α*′(cos *β*′ − 1) + sin *β*′(cos *α*′ − 1)]. The determinant must be different from zero and therefore *α*′ ≠ *pπ* and *β*′ ≠ *qπ*, being *p* and *q* integer numbers. Accordingly, the solution for the +1 frequency component can be expressed as

$$\begin{array}{c} {\hat{C}}_{+1}\left(u\right)=\frac{{\tilde{I}}_{-\alpha }\left(u\right)(e^{i\beta^{\prime }}-1)+{\tilde{I}}_{0}\left(u\right)\left(e^{-i\alpha^{\prime }}-e^{i\beta^{\prime }}\right)+{\tilde{I}}_{+\beta }\left(u\right)(1-e^{-i\alpha^{\prime }})}{i[\sin\alpha^{\prime }\left(\cos\beta^{\prime }-1\right)+\sin\beta^{\prime }\left(\cos\alpha^{\prime }-1\right)]}. \end{array}$$ (12)

Substituting the three phase-shifted images from Eq (5) in the former solution we obtain the following equation:

$$\begin{array}{c} \;{\hat{C}}_{+1}\left(u\right)=[K(\alpha^{\prime },\beta^{\prime })\cdot \tilde{O}(u-A)+L(\alpha^{\prime },\beta^{\prime })\tilde{O}(u+A)]\left|H\left(u\right)\right|. \end{array}$$ (13)

where

$$\begin{array}{c} K(\alpha^{\prime },\beta^{\prime })=\frac{{\text{e}}^{-i\alpha }-{\text{e}}^{i\alpha^{\prime }}-{\text{e}}^{i\beta }+{\text{e}}^{-i\beta^{\prime }}+{\text{e}}^{i(\alpha^{\prime }+\beta )}-{\text{e}}^{-i(\alpha +\beta^{\prime })}}{i[\sin\alpha^{\prime }\left(\cos\beta^{\prime }-1\right)+\sin\beta^{\prime }\left(\cos\alpha^{\prime }-1\right)]}\\ L(\alpha^{\prime },\beta^{\prime })=\frac{{\text{e}}^{-i\alpha }-{\text{e}}^{-i\alpha^{\prime }}-{\text{e}}^{i\beta }+{\text{e}}^{i\beta^{\prime }}+{\text{e}}^{-i(\alpha^{\prime }-\beta )}-{\text{e}}^{-i(\alpha -\beta^{\prime })}}{i[\sin\alpha^{\prime }\left(\cos\beta^{\prime }-1\right)+\sin\beta^{\prime }\left(\cos\alpha^{\prime }-1\right)]}\;. \end{array}$$ (14)

As it can be seen from the above equation, there is no contribution from the zero SI component to the +1 component estimated solution, independently of the phase-shifts values. Substituting $i_{peak}^{+1}=⎸{\hat{C}}_{+1}(+A)⎸$ and $i_{peak}^{-1}=⎸{\hat{C}}_{+1}(-A)⎸$ leads to

$$\begin{array}{c} i_{peak}^{+1}(\alpha^{\prime },\beta^{\prime })=|K(\alpha^{\prime },\beta^{\prime })\cdot \tilde{O}\left(0\right)+L(\alpha^{\prime },\beta^{\prime })\cdot \tilde{O}\left(2A\right)||H\left(A\right)|\\ i_{peak}^{-1}(\alpha^{\prime },\beta^{\prime })=|L(\alpha^{\prime },\beta^{\prime })\cdot \tilde{O}\left(0\right)+K(\alpha^{\prime },\beta^{\prime })\cdot \tilde{O}\left(-2A\right)||H\left(-A\right)|\;, \end{array}$$ (15)

Note that in a 2D-SIM set of elementary images the values of *α* and *β* are fixed. Consequently, the normalized peak intensity difference becomes a two-dimensional function that can be written as follows:

$$\begin{array}{c} \epsilon (\alpha^{\prime },\beta^{\prime })=\frac{(|\text{K}(\alpha^{\prime },\beta^{\prime })|-|\text{L}(\alpha^{\prime },\beta^{\prime })|)\left(1-\eta \right)}{(|\text{L}(\alpha^{\prime },\beta^{\prime })|+\text{K}(\alpha^{\prime },\beta^{\prime })|)\left(1+\eta \right)}\;, \end{array}$$ (16)

being $\eta =⎸\frac{\tilde{O}\left(2A\right)}{\tilde{O}\left(0\right)}⎸$. Taking into account the attenuation of high spatial-frequency content of typical samples with respect to lower frequencies, especially when considering the effect of the OTF, *η* can be neglected, that is:

$$\begin{array}{c} |\tilde{O}\left(0\right)|>>|\tilde{O}\left(2A\right)|⟶\eta <<1\;. \end{array}$$ (17)

**Fig 1:**
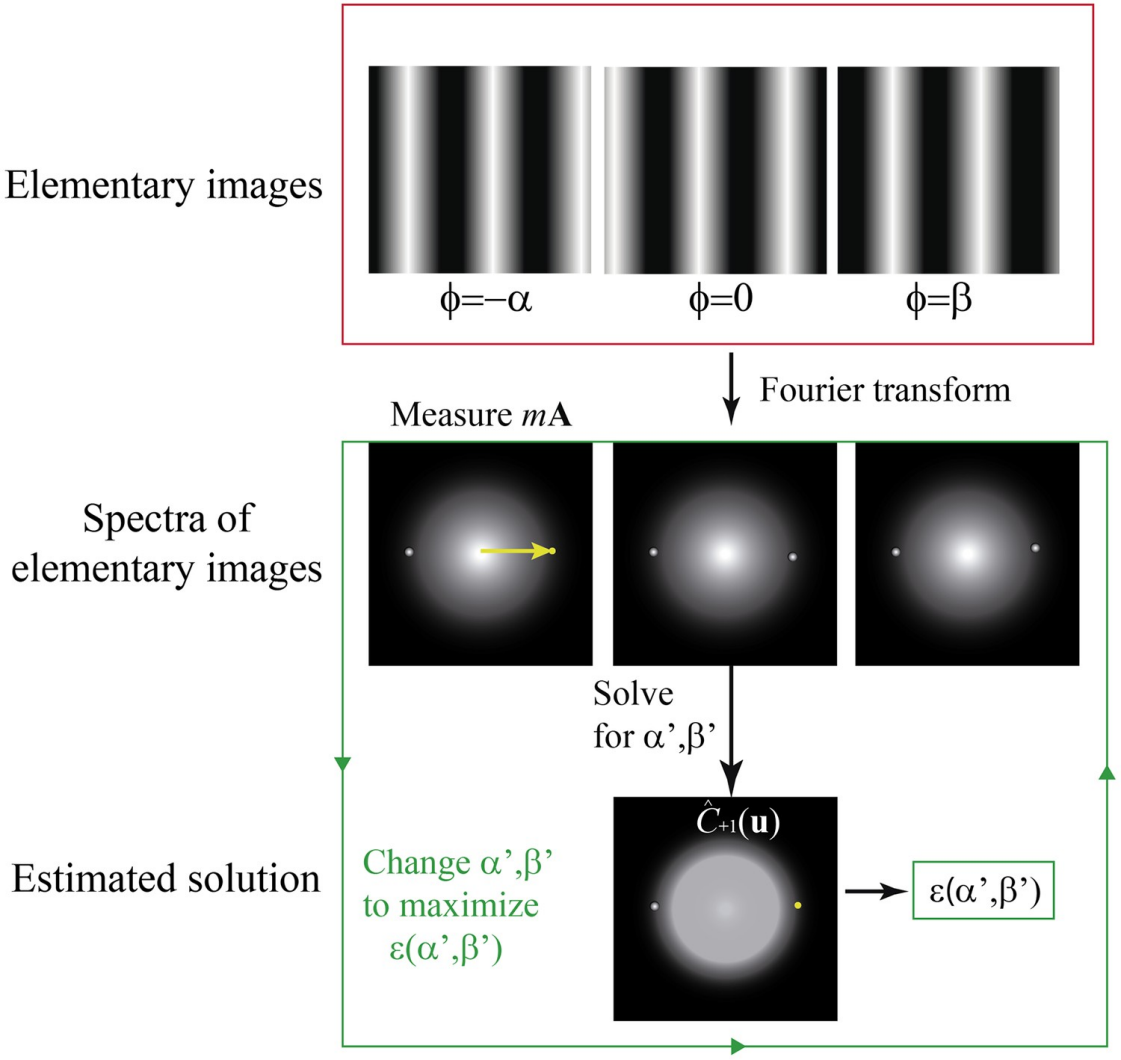
Algorithm scheme. Illustration of the normalized peak intensity difference procedure. The peak positions are measured from the Fourier transform of one elementary image. Then, the spectra of the elementary images are combined by solving the system of equations for estimated phase-shifts *α*′ and *β*′. The resulting solution represent an estimation SI components. The normalized peak intensity difference is measured and maximized through an iterative process.

**Fig 2:**
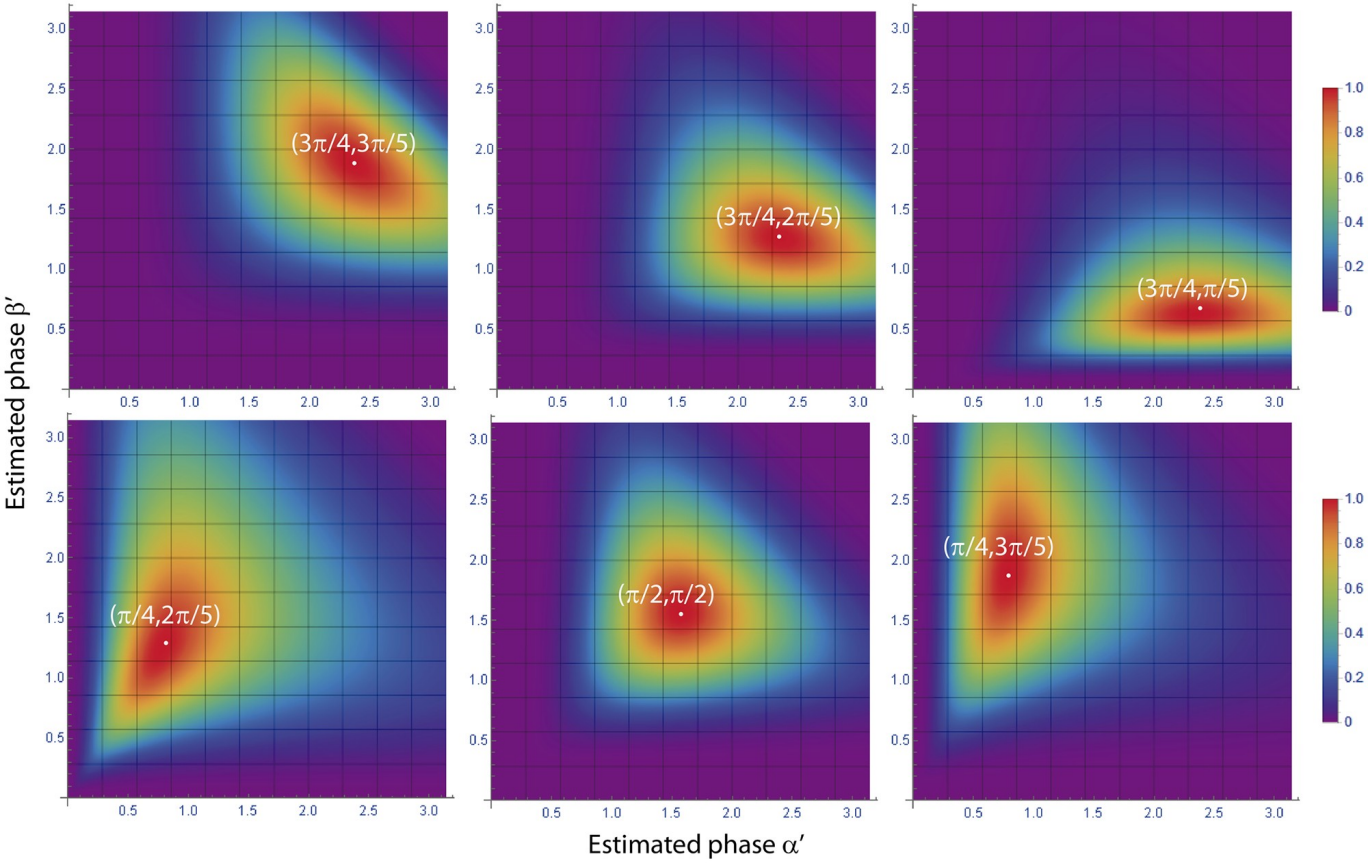
Analytical normalized peak intensity difference surfaces. Theoretical surfaces *ϵ*(*α*′, *β*′) for different real phase-shifts *α* and *β*. The marked points represent the maximum of the surface, which coincides with the estimation of the phase-shifts.

The normalized peak intensity difference can be represented as a surface for the variables *α*′ and *β*′, with a shape that depends on the experimental phase-shifts *α* and *β*. For experimental phase-shifts within a period, *α*, *β* ∈ (0, *π*), the corresponding surfaces are smooth and present an absolute maximum for *α*′ = *α* and *β*′ = *β*. This fact can be proven by calculating the maximum of *ϵ*(*α*′, *β*′) through its partial derivatives and the sign of the second derivative. We performed this task by computing the partial derivatives in Mathematica and solving the resulting system of equations. The partial derivatives were equal to zero for *α*′ = ±*α* + 2n*π* and *β*′ = ±*β* + 2n*π*, being n an integer number. It can be shown that the second derivative for those values of the estimated phase-shifts is always negative, which proves the presence of a maximum in the surface when the estimated phase-shifts match the values of the experimental phase-shifts. In practice, the total phase-shift of the SI pattern is smaller than 2*π*, which means that the multiplicity of solutions is avoided. A scheme of the proposed algorithm is illustrated in Fig 1. Surfaces for different values of the real phase-shifts calculated from Eq (16) are presented in Fig 2. The surfaces display a maximum when the estimated and experimental phase-shifts are equal to each other. Therefore, *ϵ*(*α*′, *β*′) can be considered as a metric of goodness, higher values of this function provide better estimations of the phase-shifts.

In practice, an iterative process must be implemented in order to find the maximum of the surface. In doing so, the estimated solutions are obtained for a set of increasing values of the estimated phase-shifts $\left(\alpha_{r+1}^{\prime },\beta_{s+1}^{\prime })=(\alpha_{r}^{\prime },\beta_{s}^{\prime })+(\Delta \alpha ,\Delta \beta \right)$, being *r* and *s* the iteration counters and Δ*α* and Δ*β* the phase-step. Consequently, the best estimation of the phase-shifts are the values $\left(\alpha^{\prime }=\alpha_{max}^{\prime },\beta^{\prime }=\beta_{max}^{\prime }\right)$ for which the surface presents a maximum, matching the following condition

$$\begin{array}{c} \epsilon (\alpha_{max}^{\prime },\beta_{max}^{\prime })=max(\epsilon (\alpha^{\prime },\beta^{\prime }))\Leftrightarrow (\alpha_{max}^{\prime },\beta_{max}^{\prime })=(\alpha ,\beta )\to {\hat{C}}_{m}^{j}=C_{m}^{j}\;. \end{array}$$ (18)

**Fig 3:**
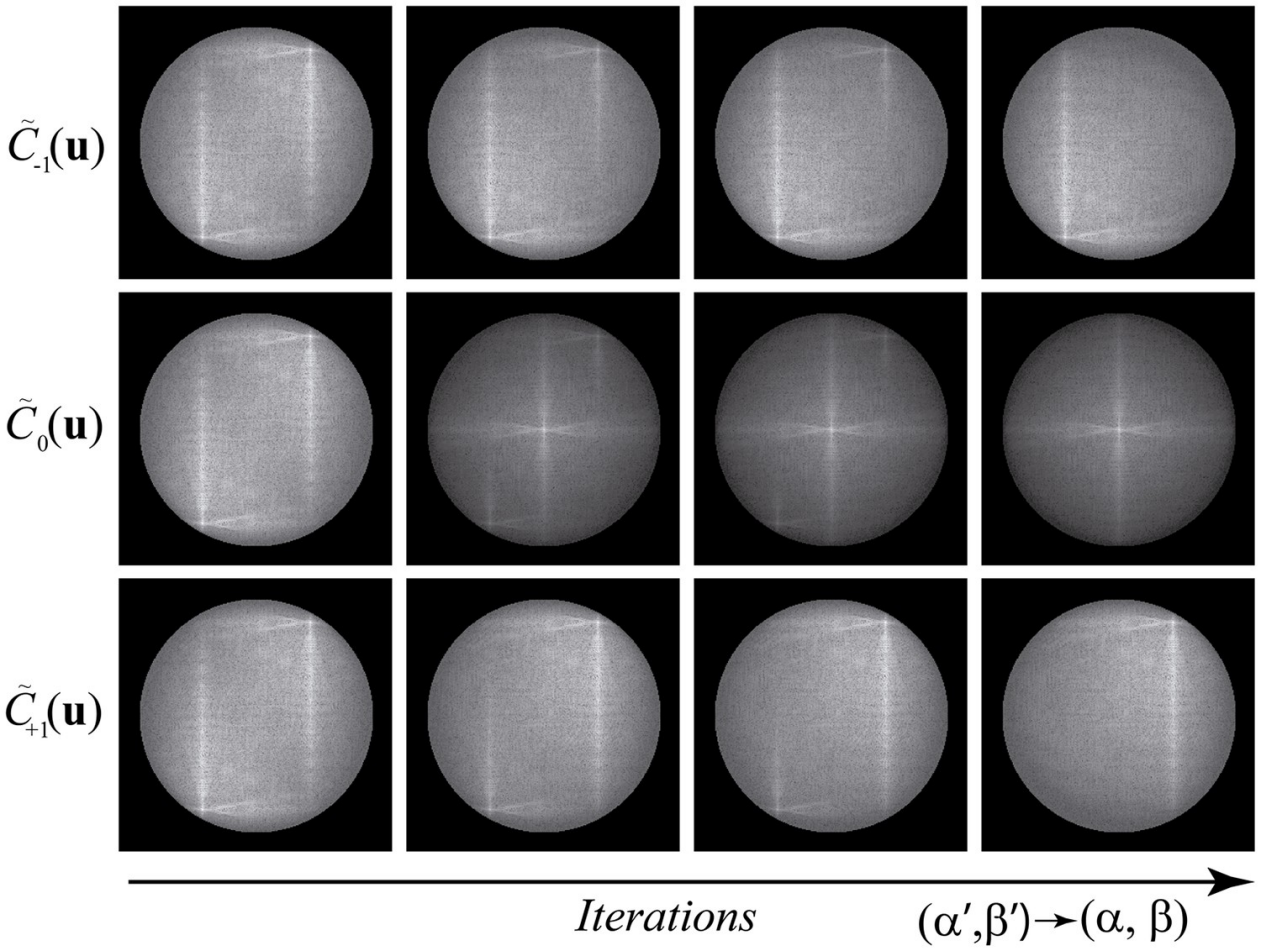
Method convergence illustration. Example of the convergence of the solutions as the value of the estimated phase-shifts approach the experimental phase-shifts.

The precision of the estimation depends on the values of Δ*α* and Δ*β* as well as the statement in Eq (18) is only true for Δ*α* = Δ*β* → 0. An illustration of the convergence of the algorithm is shown in Fig 3. Note that the peak intensities of the SI components are constant so they must be measured once. Hence, the resulting normalized peak intensity difference function depends exclusively on the phase-shift estimates. As a result, instead of calculating every point of the surface *ϵ*(*α*′, *β*′) with a given accuracy, a nonlinear programming solver (BFGS quasi-Newton algorithm [21]) was used to find the minimum of 1/*ϵ*(*α*′, *β*′). The nonlinear optimization drastically reduces the computation time of the proposed method, providing precise phase-shift estimations in the order of milliseconds. In a whole reconstruction process, the proposed approach must be applied to 3 angles of SI pattern. The resulting isolated spatial-frequency components are recombined by means of Eq (9) and then Fourier transformed to obtain the 2D-SIM reconstructed image.

## Results and discussion

### Simulation

**Fig 4:**
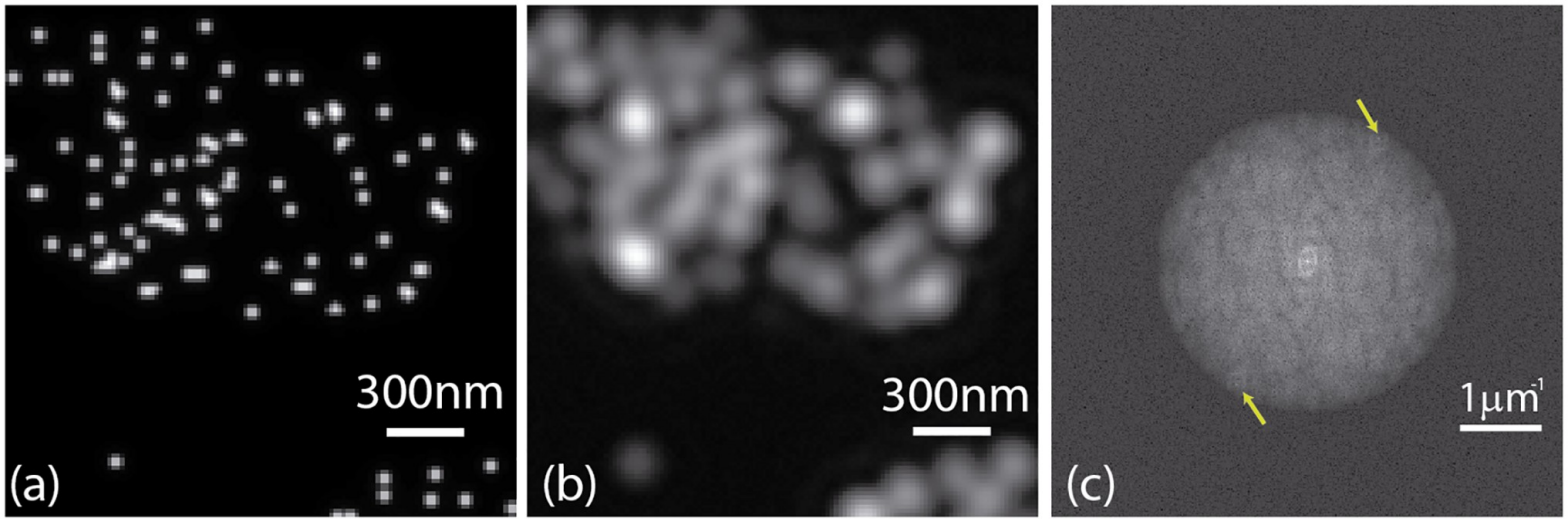
Simulation of a structured illumination microscope imaging process. (a) Synthetic object consisting of a cluster of beads of size 5 times smaller than the wide-fiield point spread function. (b) Simulated image intensity distribution of an elementary image of the synthetic object and (c) its corresponding Fourier transform (modulus). The arrows show the position of the peak of the SI components.

**Fig 5:**
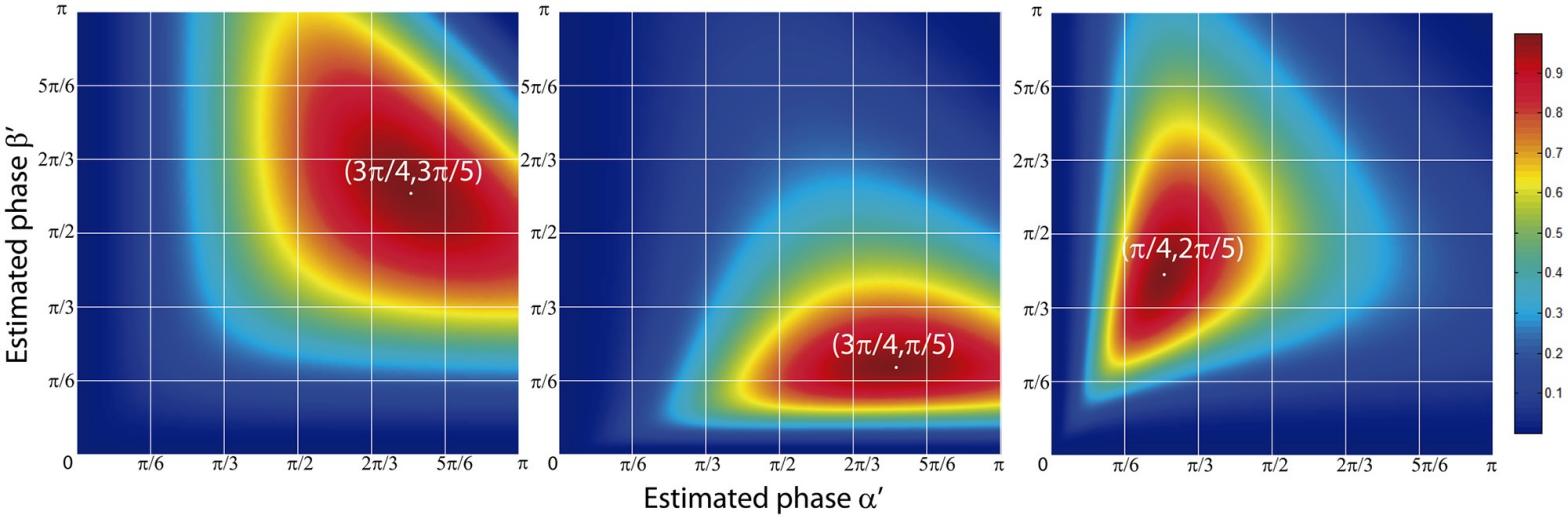
Simulated normalized peak intensity difference surfaces. *ϵ*(*α*′, *β*′) surfaces calculated from the simulation for different phase-shifts. The surfaces present an absolute maximum when the position of the estimated phase-shifts match the phase-shifts used as inputs.

In this section, we test the proposed method by means of a simulation. To this end, we firstly made a convergence study of the normalized peak intensity depending on the experimental phase-shifts. Afterward, we used this study to propose a time-optimized iterative algorithm to estimate the values of the unknown phase-shifts. The performance of our method is compared to the standardly used algorithms in terms of the error in the phase-shifts estimation. We simulated the image formation through a 2D-SIM system with experimental parameters of NA = 1.4, and emission wavelength equal to 512nm. We generated an object consisting of a cluster of beads of size 5 times smaller than the wide-field 2D-PSF, see Fig 4(a). After that, a SI pattern with a spatial frequency of 95% of the cut-off frequency of the wide-field system was simulated and projected onto the object, see Fig 4. The resulting spectrum was filtered by a simulated 2D-OTF and Fourier transformed to generate the simulated image intensity distribution, as described in Eq (3). In this simulation phase-shifts can be chosen to generate the three elementary images required in the 2D-SIM reconstruction and then compared with the estimated values calculated by the normalized peak intensity difference. In addition, we considered the presence of Gaussian and Poisson noises by using the following imaging model [22]:

$$\begin{array}{c} I(\left(x\right)=g[I_{p}\left(x\right)+n_{p}\left(x\right)]+n_{r}\left(x\right)\;, \end{array}$$ (19)

being *g* the gain of the sensor, *Ip*(**x**) is the expected number of photons of the image, *n_p_*(**x**) the photon noise and *n_r_*(**x**) the readout noise of the camera. The gain was set to 1 in order to simplify the analysis. Thus, elementary images in terms of the number of photons are calculated by means of Eq (19). All simulated images are affected by shot noise as well as a maximum of 10 photons of gaussian readout noise. In order to verify the validity of the normalized peak intensity difference method, we simulated the acquisition of three sets of elementary images with a maximum expected number of photons *n*_*max*_ = 2000 and for phase-shifts equal to $\left(\frac{3\pi }{4},0,\frac{3\pi }{5}\right)$, $\left(\frac{3\pi }{4},0,\frac{\pi }{5}\right)$ and $\left(\frac{\pi }{4},0,\frac{2\pi }{5}\right)$. A zoomed-in region of one of the simulated elementary images and its corresponding Fourier transform are shown in Fig 4. Simulated data were used as input in the proposed phase-shift estimation algorithm. The peak positions of the SI components were obtained by applying a conventional peak detection to one of the elementary images Fourier transform. Note that the accuracy of this detection can be improved by zero-padding the elementary images [16]. We calculated the surface *ϵ*(*α*′, *β*′) for each simulated acquisition with steps equal to Δ*α* = Δ*β* = 10^−4^. The resulting surfaces are presented in Fig 5. The surfaces match the shape predicted by theory, see Fig 2 for comparison. Furthermore, the absolute maximum of the surfaces corresponds to the estimated phases. In order to prove it, we measured the values of the maximum of each surface and compared them to the simulated phase-shifts. As a result, the estimated phase-shifts matched the simulated values, with an accuracy *σ*(*α*), *σ*(*β*) strictly dependent on Δ*α* and Δ*β* (see values in Table 1).

**Table 1:** Values of the estimated phase-shifts (*α*′, *β*′) calculated from the normalized peak intensity surface for different values of the phase-steps Δ*α*, Δ*β*. All values are expressed in radians.

Δ*α*, Δ*β*	*α*	*α*′	*σ*(*α*)	*β*	*β*′	*σ*(*β*)
0.01	3*π*/4	2.36	0.01	3*π*/5	1.88	0.01
0.001	3*π*/4	2.356	0.001	3*π*/5	1.885	0.001
0.0001	3*π*/4	2.3562	0.0001	3*π*/5	1.8849	0.0001

**Fig 6:**
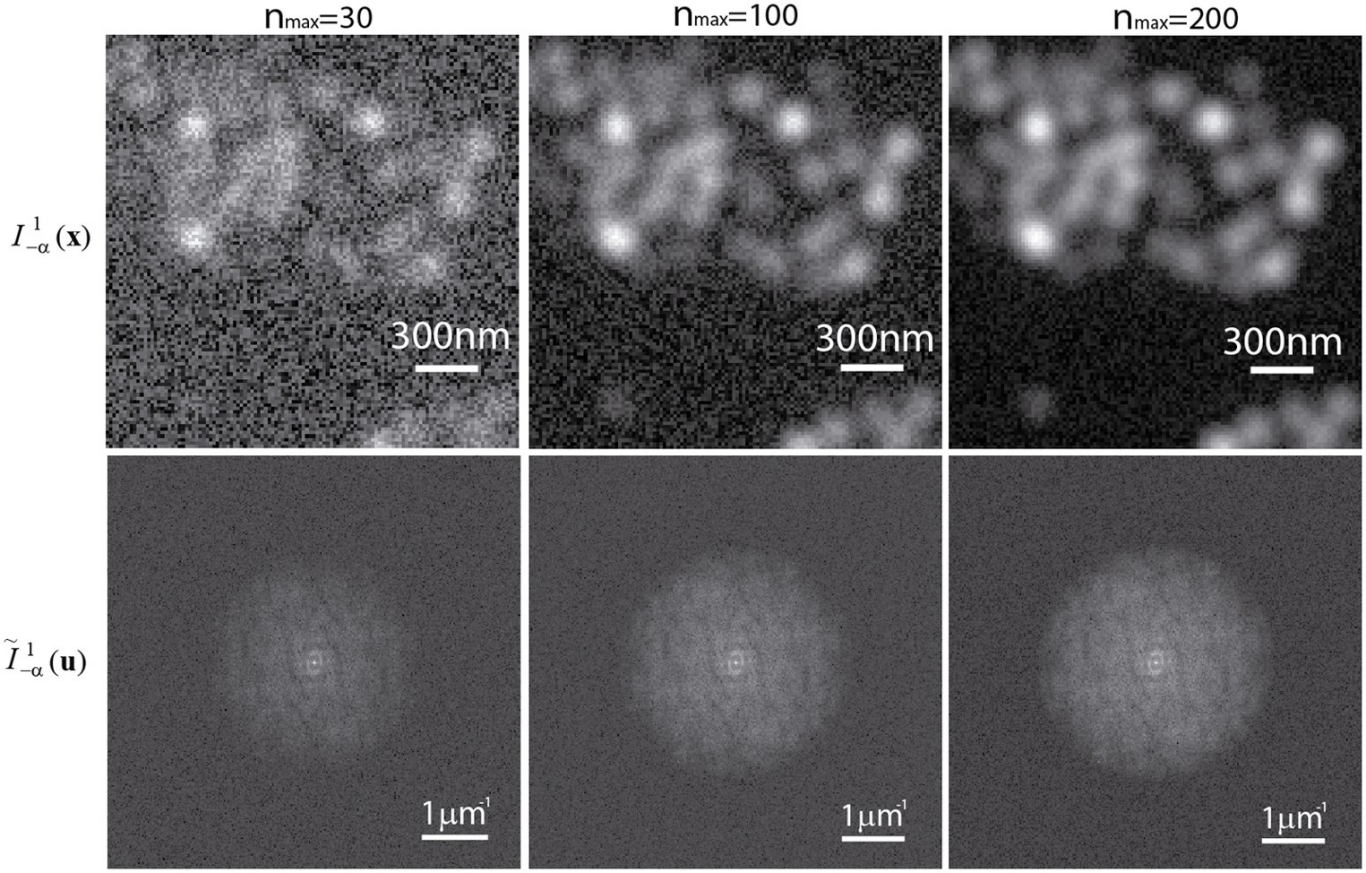
Simulated elementary images in presense of noise. Intensity distributions of an elementary image of the simulated cluster of beads for different maximum expected number of photons (*n*_*max*_ = 30, 100, and 200) (top row), their corresponding spectra (bottom row).

**Fig 7:**
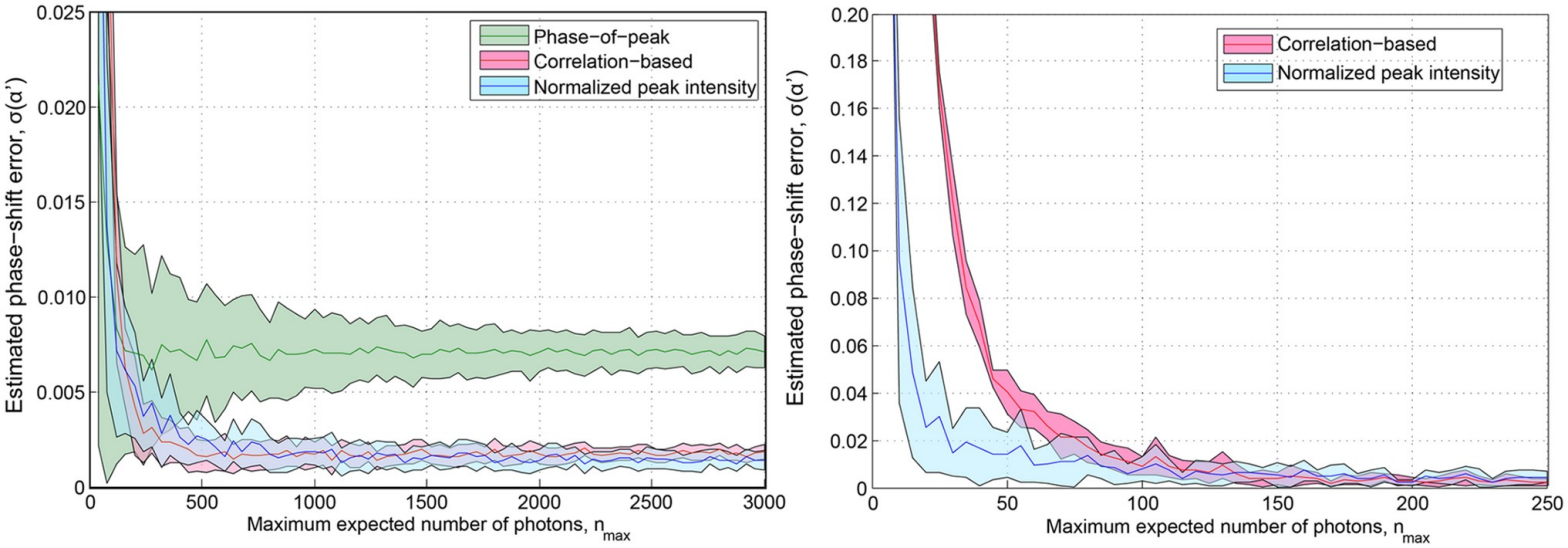
Tolerance to noise comparison. Comparison of the estimated phase error as a function of the maximum expected number of the photons for the phase-of-peak, iterative cross-correlation, and the proposed method. In addition to the shot noise a read-out Gaussian noise of 10 photons was added to the simulated images. The coloured areas represent the standard deviation of errors.

**Fig 8:**
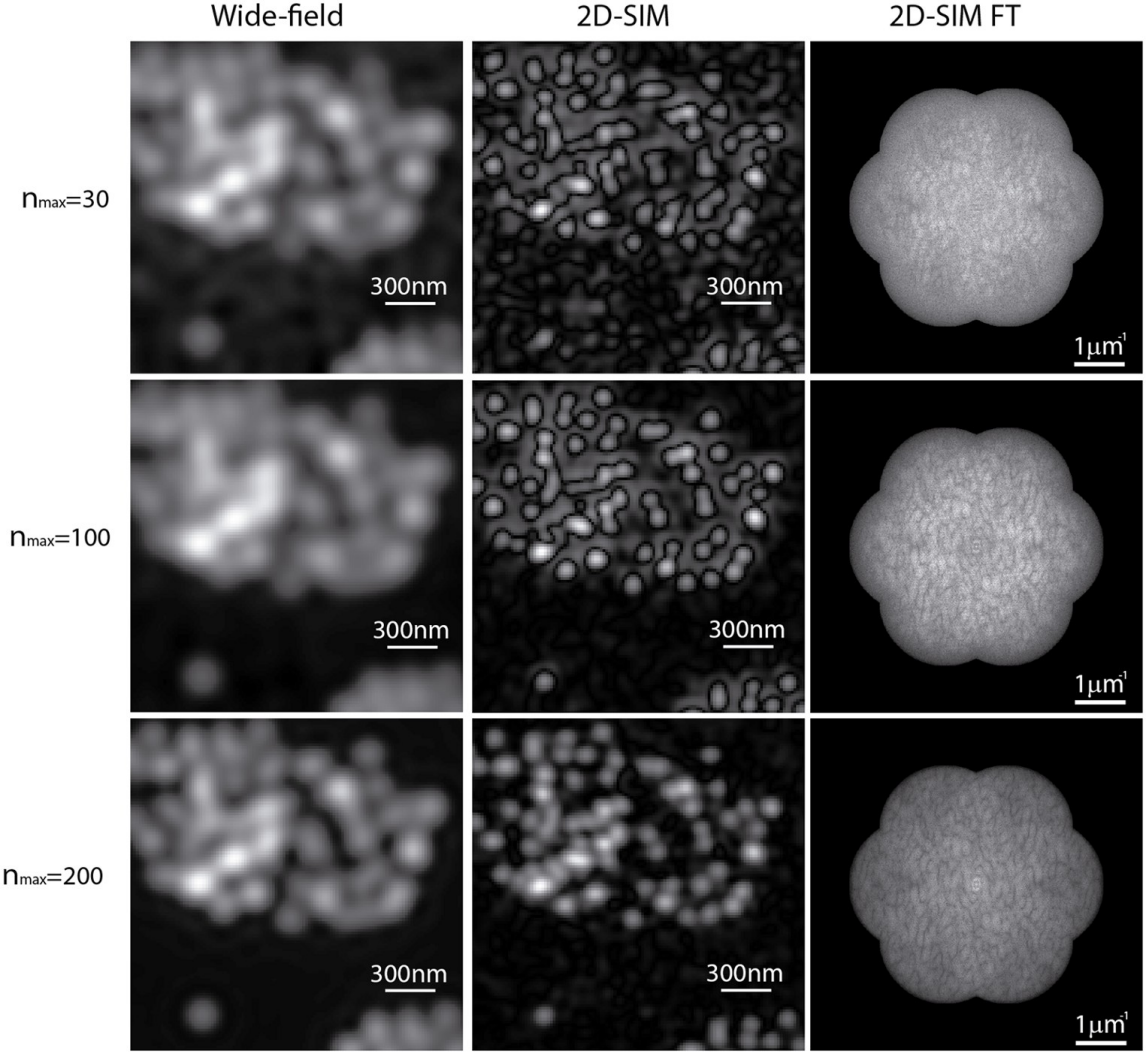
Simulated wide-field and 2D-SIM images depending on the maximum expected number of photons. Comparison between the simulated wide-field images and the 2D-SIM reconstructed images for different values (*n*_*max*_ = 30, 100, and 200) of the maximum expected number of photons. The spectra of the 2D-SIM reconstructed image is also presented. Although the images are highly affected by noise, in the worst case (*n*_*max*_ = 30) the algorithm still provided an estimation of the phase-shifts with an error smaller than 5%.

In a next step, we changed the maximum expected number of the photons, *n*_*max*_, collected by the optical system for assessing the effect of the presence of noise in the simulation. Examples of simulated elementary images in low-photon conditions (*n*_*max*_ = 30, 100, and 200) and their corresponding Fourier transforms are shown in Fig 6. We also implemented the iterative cross-correlation algorithm [18] and the method based on the measurement of the phase from the peak components [19] to compare the performance with our method. We applied the three algorithms varying the maximum expected number of photons from 10 to 3000 by randomly adding shot-noise and a maximum of 10 photons of Gaussian read-out noise. At that point, we calculated the error in the determination of the phase-shifts by comparing the estimated phase-shifts with the inputs. This measurement was repeated 30 times for each value of *n*_*max*_. An average of the 30 estimated phase-shift errors as well as the standard deviations are represented in the left plot of Fig 7. As expected, the error in the phase-shift estimation for the phase-of-peak method was relatively high compared to the correlation-based and the normalized peak intensity difference. Furthermore, the precision of the phase-of-peak algorithm remains constant, at ranges of 10^−2^ rad. On the other hand, the correlation-based and the proposed method showed a similar behaviour leading to errors in the order of 10^−3^ rad for relatively low levels of maximum expected photons, *n*_*max*_ > 500. In addition, a zoomed-in region of the plot is presented in the right plot of Fig 7 in order to compare the performance of the proposed method and the correlation-based in low-photons conditions. Results indicate that both algorithms performed with the same accuracy for a maximum expected number of photons *n*_*max*_ > 100. The proposed method provided relatively accurate estimations of the phase-shifts even when the relationship between the expected number of photons and the noise was significantly low (*n*_*max*_ = 30). Whole reconstruction examples performed with the proposed method in low SNR conditions for three angles of the projected pattern (−60°, 0°, 60°) are presented in Fig 8. In such cases, the reconstructed images were highly affected by noise, but the proposed algorithm was efficient to decompose and shift the SI components.

**Fig 9:**
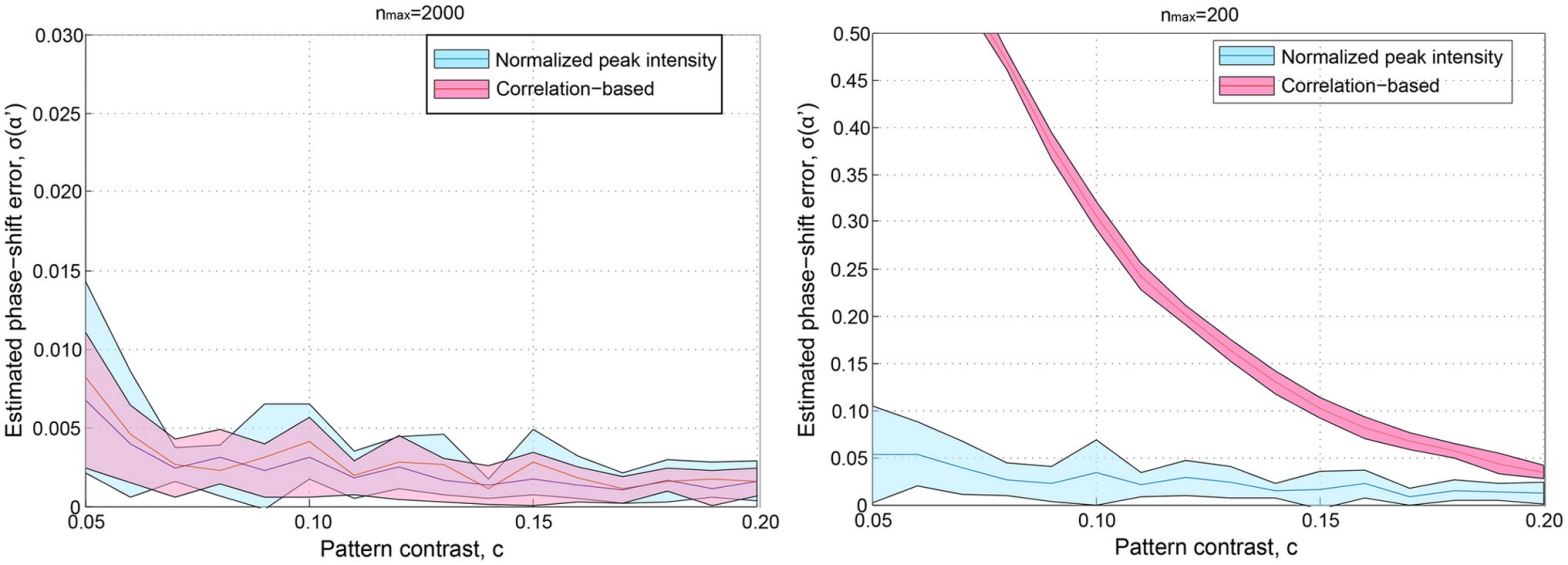
Tolerance to the pattern contrast comparison. Average phase-shift estimation errors as a function of the pattern contrast for maximum expected number of photons equal to 200 and 2000. The filled area represent the standard deviation of the 30 measurement performed for each value of the contrast. In addition to shot noise, a readout noise of 10 photons was added to the images.

**Fig 10:**
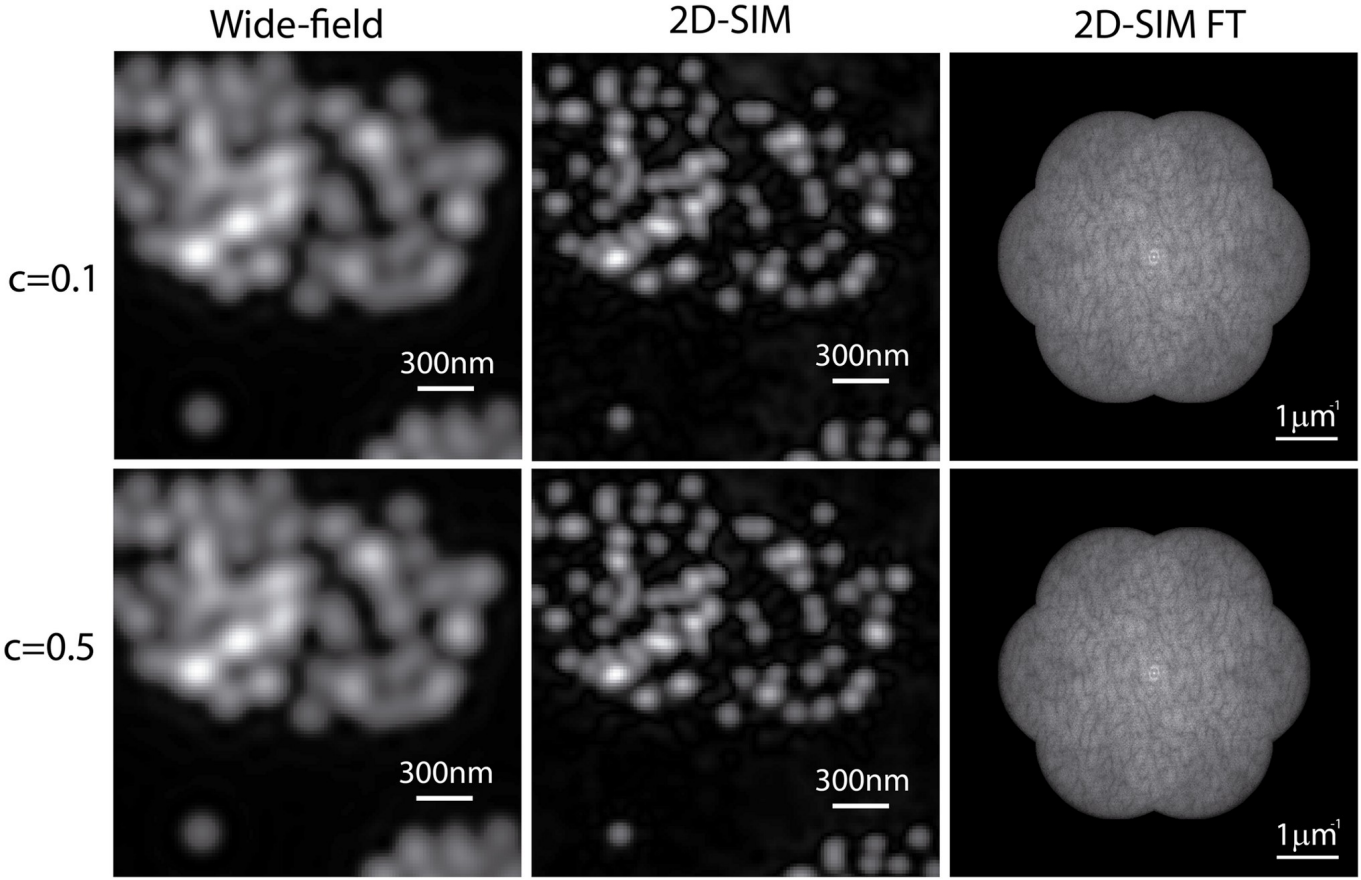
Tolerance to the pattern contrast. Simulated wide-field and 2D-SIM for contrasts of the SI pattern equal to 0.1 and 0.5. The maximum expected number of photons was set to 200 photons, with a maximum of 10 photons of Gaussian noise in addition to the shot noise. Even in these unfavorable conditions, the algorithm provided an estimation of the phase-shifts with an error smaller than 1%. As expected, the resulting reconstructions are affected by noise but the unmixing of the components was properly achieved by the proposed method.

Until now, we have considered an ideal SI pattern of contrast equal to 1. In practice, this value can be affected by the coherence of the light source, by an unequal power distribution of the intefering beams, or by any light source from out-of-focus planes. The proposed approach is based on the detection of the peaks produced by the SI in the Fourier domain, so it is relevant to examine the behavior of the algorithm in terms of the pattern contrast. Sets of 2D-SIM data were simulated form several values of the pattern contrast and maximum expected number of photons, maintaining the corresponding level of noise (10 photons of standard deviation). We compared the error in the phase-shifts estimation of the proposed method with the cross-correlation algorithm. Results showed that both algorithms estimated the phase-shifts with the same precision for values of the contrast higher than 0.2 and expected number of photons equalt to 200 and 2000. However, the error in the phase-shift estimations started to increase for pattern contrasts smaller than 0.2. Curves displaying the behavior of both algorithms as a function of the pattern contrast and maximum expected number of photons are shown in Fig 9. The curves show the average phase-shift estimation over 30 images for each value of the contrast, affected by different random noises as well as the standard deviation of the measurements. Nevertheless, even under extremely unfavorable conditions such as *c* < 0.1, an estimation of the phase-shifts with errors below 1% were calculated by both methods for *n*_*max*_ = 2000. Additionally, the correlation-based algorithm showed less tolerance than the proposed method when the contrast of the pattern was below 0.2 for *n*_*max*_ = 200. Examples of the whole reconstruction carried out with our method are presented in Fig 10, for *n*_*max*_ = 200 and pattern contrasts 0.1 and 0.5. Both reconstructions present the expected enhancement in the lateral resolution with a more significant influence of the noise for a more diminished pattern contrast.

In order to provide an estimation of the computation time, we performed an estimation of 100 random values of the phase-shift for a single direction of the pattern. For a tolerance of 10^−6^ the average computation time was (0.0070±0.0005)s. The calculations were performed in Matlab by means of a computer with an Intel Core-i7-3770 3.4 GHz processor with 20 GB of RAM.

We can conclude that in practical conditions (a high expected number of photons, low noise and high contrast of the pattern) the proposed method and the iterative correlation based algorithm [18] present the same performance in terms of the accuracy. In that sense, the normalized peak intensity difference represents a computational simplification of the method proposed by Wicker et al. Furthermore, the presented method can be optimized reducing the computation time to few milliseconds. Nevertheless, the main drawback of our method is that the peaks of the SI components need to be measurable: they must be inside the band support of the OTF as well as they must have enough photons to be detected.

### Experimental results

In this section, we tested the proposed method by using experimental data. For that purpose, we built a structured illumination microscope consisting of a fiber-coupled laser (λ = 488*nm*), two optical relays of magnification *M*_1_ = 1 and *M*_2_ = 1, a MO Olympus M = 50x, NA = 0.50, a tube-lens (TL) of focal length 200mm, and a sCMOS camera Hamamatsu OrcaFlash 4.0 with 2048x2048 pixels of 6.5*μ*m pixel size. The structured illumination is produced by a phase diffraction grating of 50 lp/mm (Pasco). The zero-order of the grating was blocked out by using a spatial filter (located at the image plane of the first relay system) whilst the higher orders were filtered by the MO aperture stop, which were projected by means of the second optical relay. Since the grating was illuminated by a spherical wave, the separation of the orders could be tuned by changing its distance to the point source, which allowed the projection of a SI pattern of the desired spatial frequency [23]. The grating was mounted onto a rotating plate held by a stepper motor (Owis) to produce the rotations of the pattern and the transverse movement required for the phase-shifting the SI pattern, respectively. A long-pass filter (*l*_*cut*_ = 530*nm*) was used to filter the laser light in the collection whilst allowing the fluorescent light to reach the camera.

**Fig 11:**
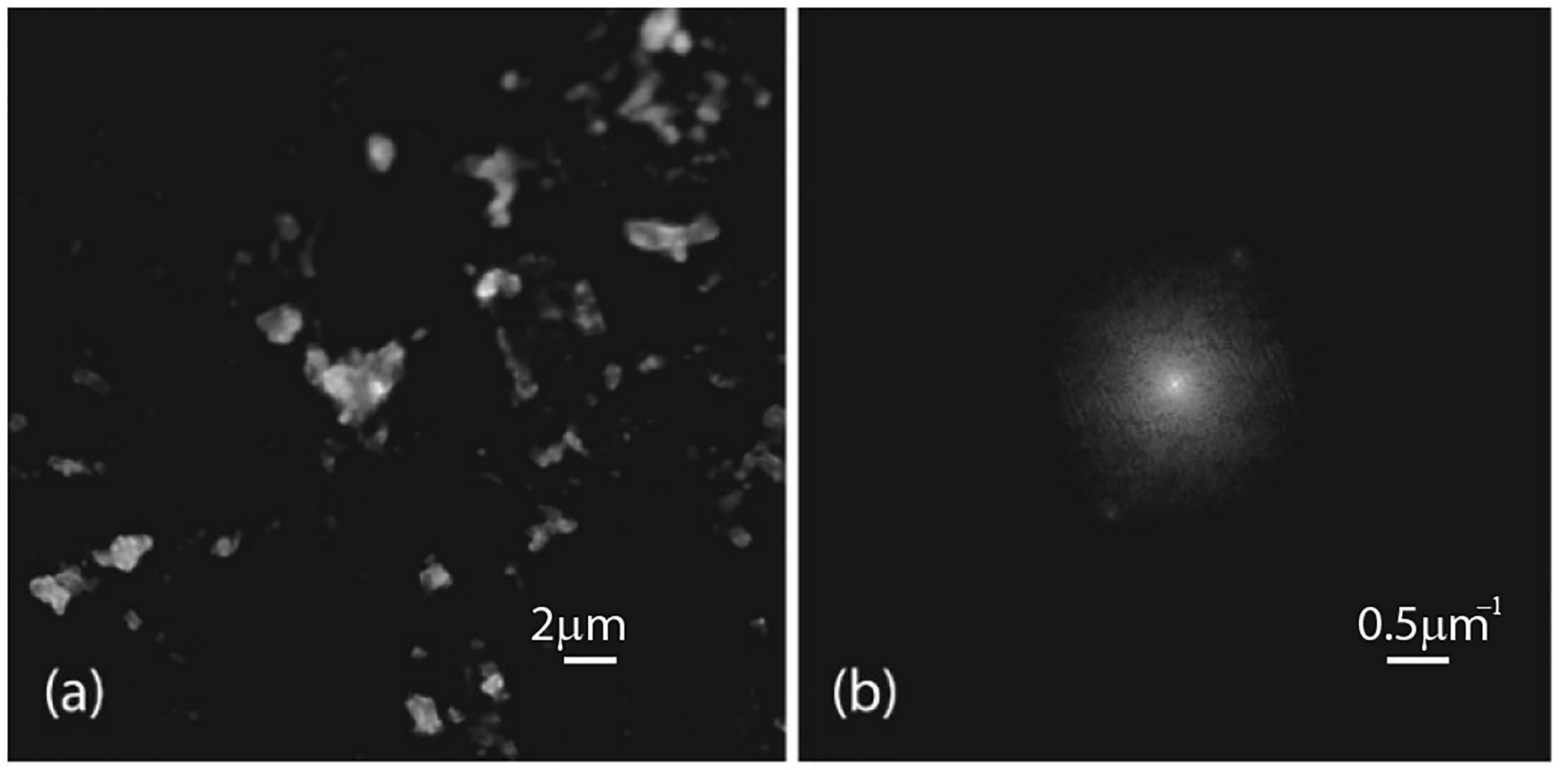
Elementary experimental image. (a) Elementary image of a dye solution of cotton fibers and (b) its corresponding Fourier transform.

**Fig 12:**
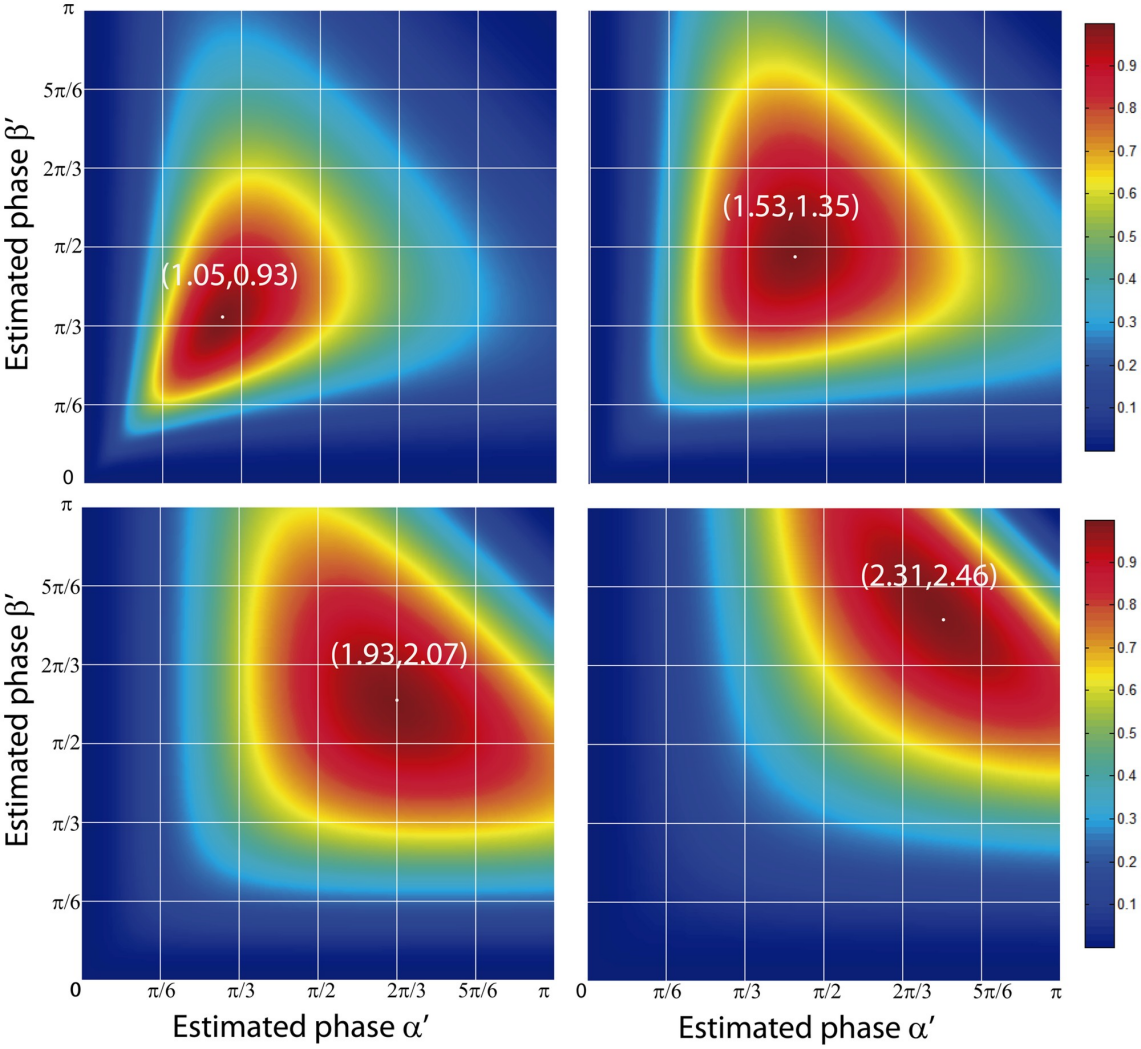
Normalized peak intensity difference surfaces for different values of the phase-shifts. Normalized peak intensity difference surfaces obtained for various sets of elementary images. The phase-shifts were generated by displacing the diffraction grating a given amount of steps with the stepper motor. The surfaces show the expected behavior, that is, the phase-shifts proportionally increased with the number of steps.

With this setup, we tested the validity of the method by using a fluorescent solution of cotton fibers dyed with Rhodamine 123 as sample. We collected sets of 2D-SIM elementary images for one direction of the pattern. The experimental phase-shifts were sequentially increased. To this end, the displacement of the diffraction grating was set to an increasingly higher number of steps of the stepper motor. An elementary image captured by the system and its correspondent Fourier transform are shown in Fig 11. For each set, we calculated the normalized peak intensity surfaces, see Fig 12. Note that the contrast of the pattern (see panel (b) of Fig 11) was relatively low. We measured a contrast of 0.27 by comparing the weight of the spectral components.

**Fig 13:**
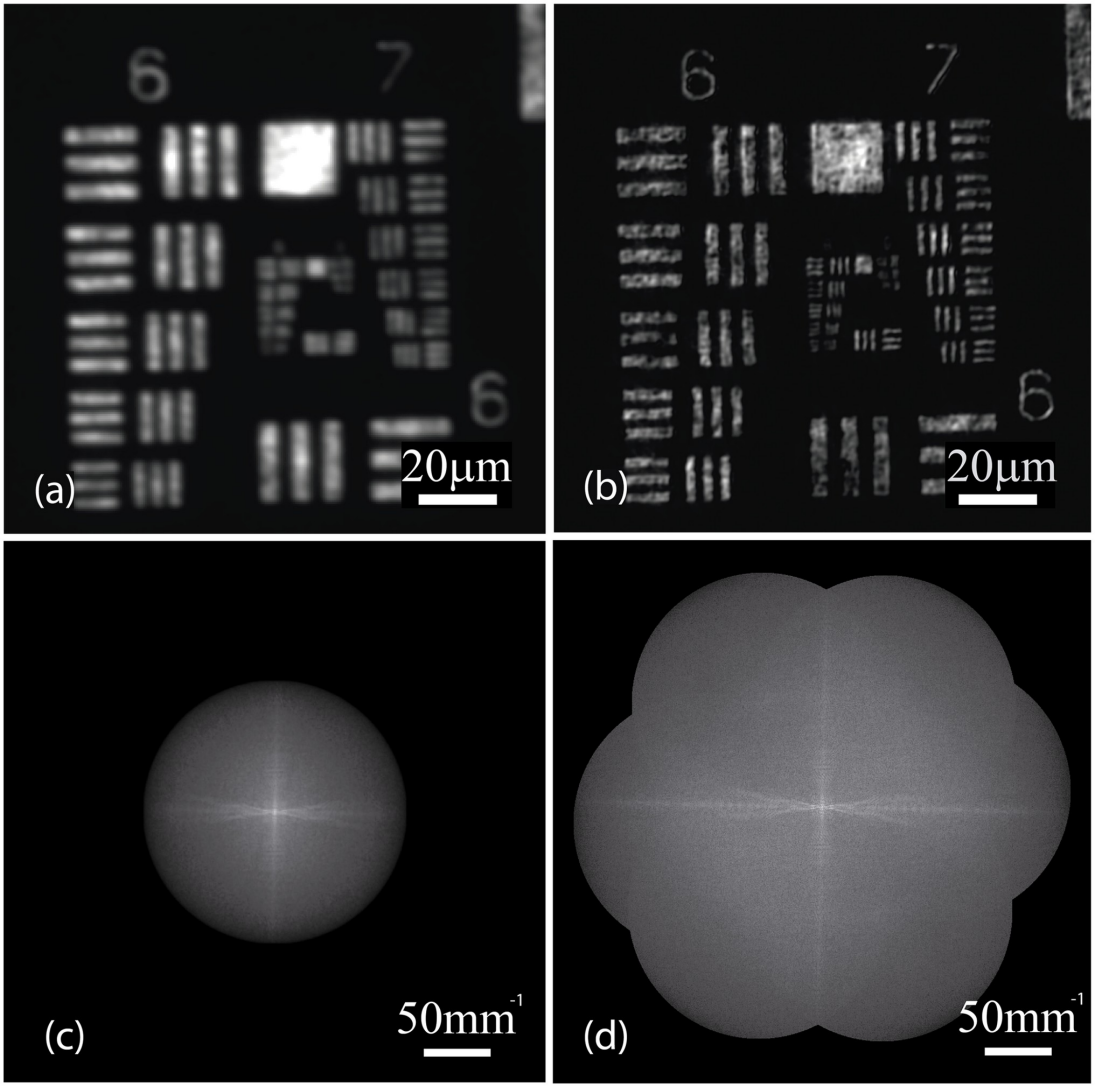
Experimental 2D-SIM reconstruction. Experimental images of a fluorescent USAF 1951 test target (NA = 0.1). The expected lateral resolution enhancement is observed between (a) the wide-field image and (b) the 2D-SIM reconstruction. Equivalently, the 2D-SIM system provided an extended bandwidth as displayed in the corresponding Fourier transforms, (c) and (d). Note that no residual peaks are displayed in (d), which means that the proposed approach produced a properly unmixed spectral components.

To demonstrate the resolution improvement provided by our system as well as validating our method in practical conditions, we carried out a proof-of-concept experiment with low numerical aperture. In this case, we used a 4x and NA = 0.1 microscope objective and a fluorescent 1951 USAF test target as sample. The spatial frequency of the SI pattern was set at approximately 92% of the cut-off frequency of the microscope objective. In these conditions, we carried out a whole 2D-SIM reconstruction by using the normalized peak intensity difference in order to estimate the phase-shifts. In this case, we applied the time-optimized algorithm to a set of elementary images with unknown phase-shifts of the projected pattern. The relative rotation angles of the diffraction grating were (−60°, 0°, 60°). The 2D-SIM reconstruction and its comparison with the wide-field image are shown in Fig 13. As it can be observed, there are no artifacts in the 2D-SIM estimated spectrum. Consequently, the estimation of the phase-shifts provided by our method is close to the experimental phase-shifts. An improvement of 5 groups can be measured in the SIM reconstruction with respect to the wide-field image, which means a resolution enhancement of a factor 1.8. Note that non-uniformities in the fluorescent dye of size close to the resolution limit become more noticeable in the SIM image due to the improvement in the lateral resolution.

**Fig 14:**
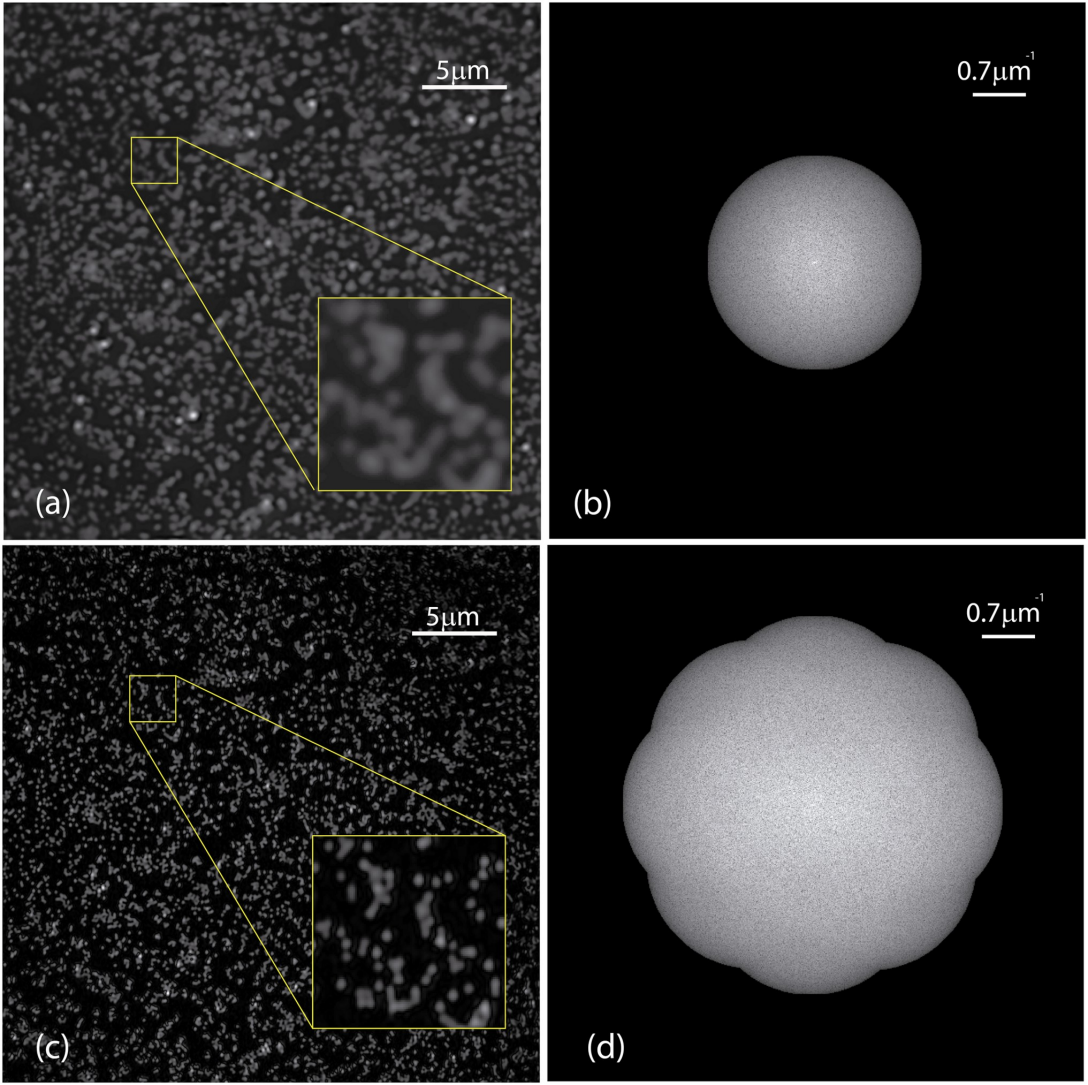
Reconstruction performed using the proposed method for a FairSIM dataset. (a) Wide-field image and (c) structured illumination images of 200 nm Tetraspeck beads. The corresponding Fourier transforms are presented in (b) and (d).

In a last experiment, we used a dataset (SLM-SIM 200nm-Tetraspeck 680nm) from the open-source FairSIM [24] repository to prove the performance of the normalized peak intensity difference for data with unknown acquisition parameters. In this dataset, four angles of the illumination pattern were used. The proposed method calculated properly the estimated phase-shifts as there is no presence of artifacts neither in the image or its Fourier transform, see Fig 14. In the same way, the expected transverse resolution improvement in the reconstructed SIM image can be observed.

## Conclusion

We have presented an alternative method for estimating the phase-shifts between elementary images in 2D-SIM. The method is based on the comparison of the peak intensities of the spatial frequency components resulting from the estimated solutions obtained with standard SIM reconstruction. The calculation of the normalized intensity difference of the peaks results in a two dimensional function with the estimated phase-shifts as coordinates. The surface presents an absolute maximum when the estimated phase-shifts are equal to the experimental ones. Thus, maximizing the intensity difference of the peaks guarantees a proper estimation of the experimental phase-shift. We showed the validity of the method via simulations and experiments. The computation time of the method can be optimized by using a nonlinear programming solver. Furthermore, we studied the stability and robustness of the results provided by the method under different conditions, such as the dependence on the phase-shift, the contrast and the presence of noise. The algorithm shows a high immunity to noise as well as to SI patterns with low-contrast. The method shows a precision in the estimation of the phase-shifts comparable to the standardly used cross-correlation method, but requiring for fewer computations and converging with a faster computation time.

## Supporting information

S1 Raw Data This folder contains all raw data and scripts for Matlab and Mathematica underlaying the manuscript. (ZIP)
