# Supplementary figures and images for: Fast and robust phase-shift estimation in two-dimensional structured illumination microscopy
Source: PLoS One. 2019 Aug 16;14(8):e0221254. doi: 10.1371/journal.pone.0221254 (PMC6697343; doi:10.1371/journal.pone.0221254)

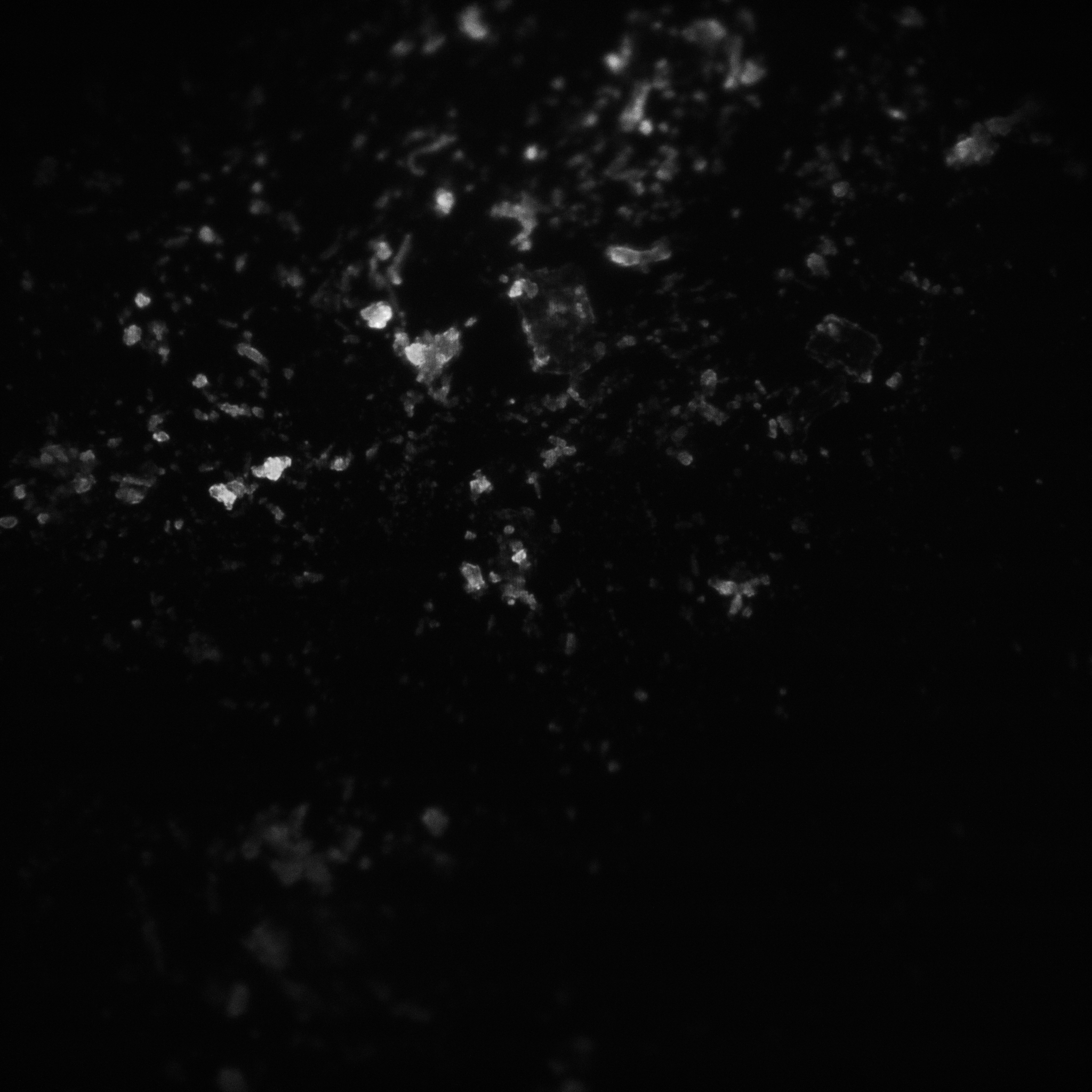

Supplement: S1 Raw Data — (ZIP) [file pone.0221254.s001.zip › Supporting_Information/fig11_motor_step/1_330.png]

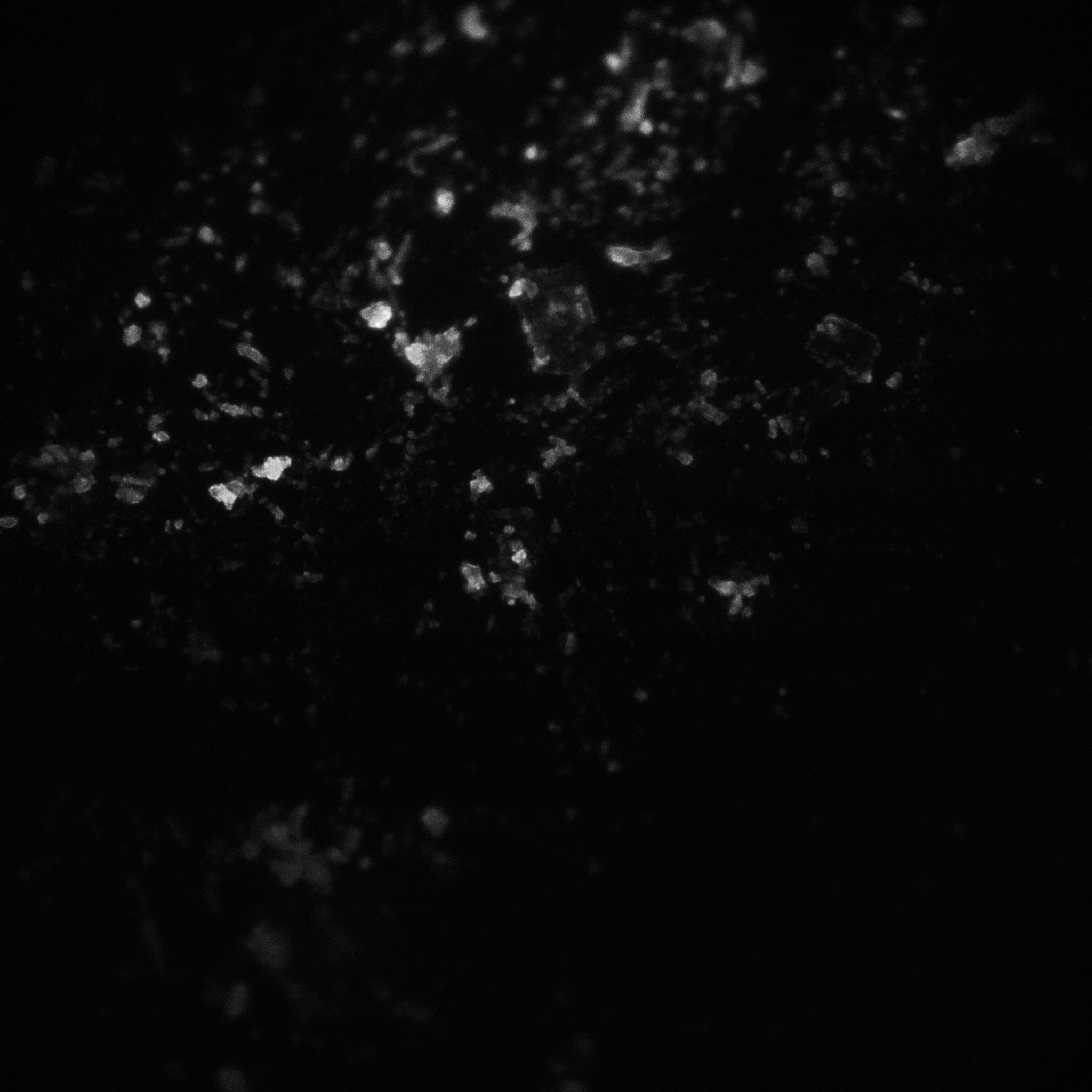

Supplement: S1 Raw Data — (ZIP) [file pone.0221254.s001.zip › Supporting_Information/fig11_motor_step/1_340.png]

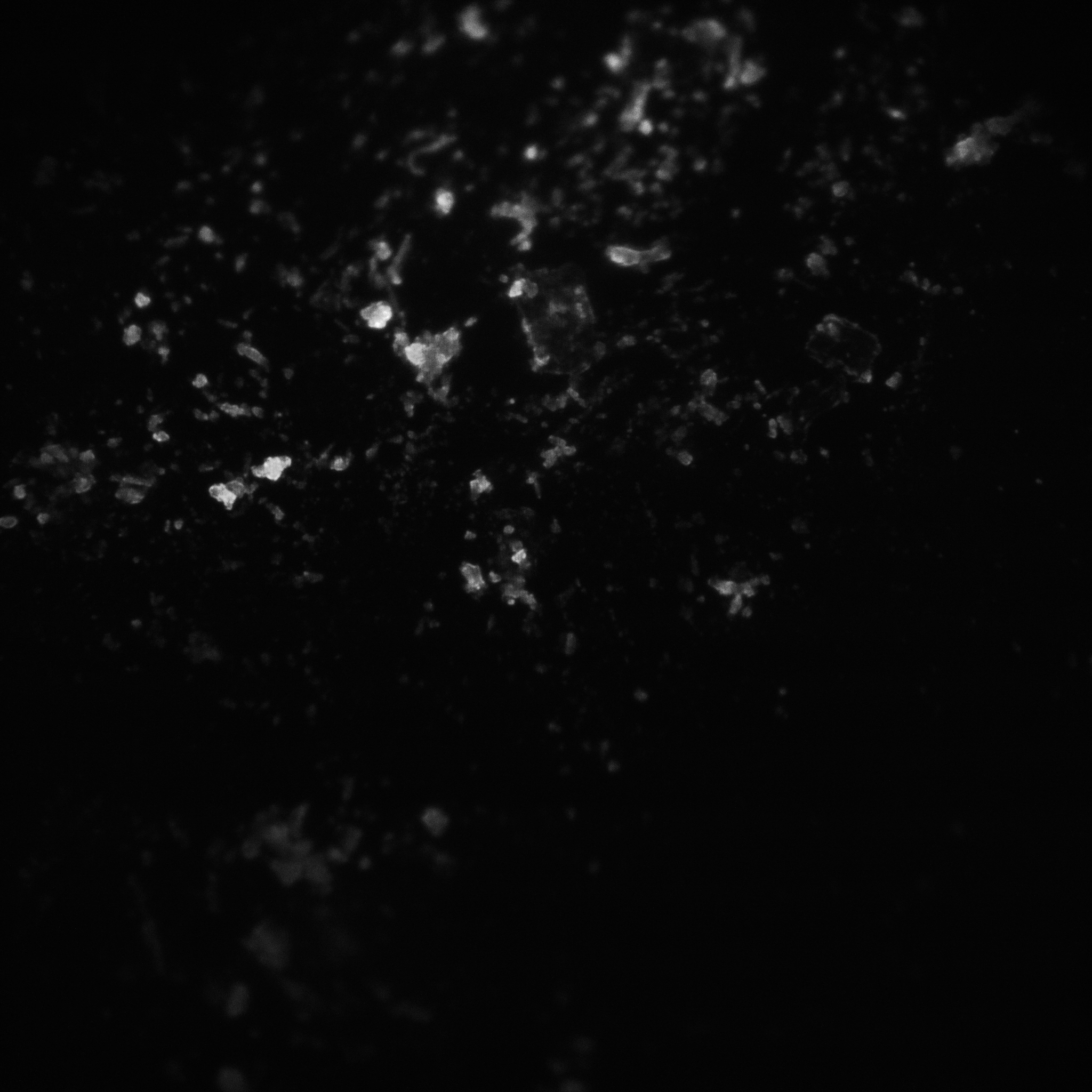

Supplement: S1 Raw Data — (ZIP) [file pone.0221254.s001.zip › Supporting_Information/fig11_motor_step/1_350.png]

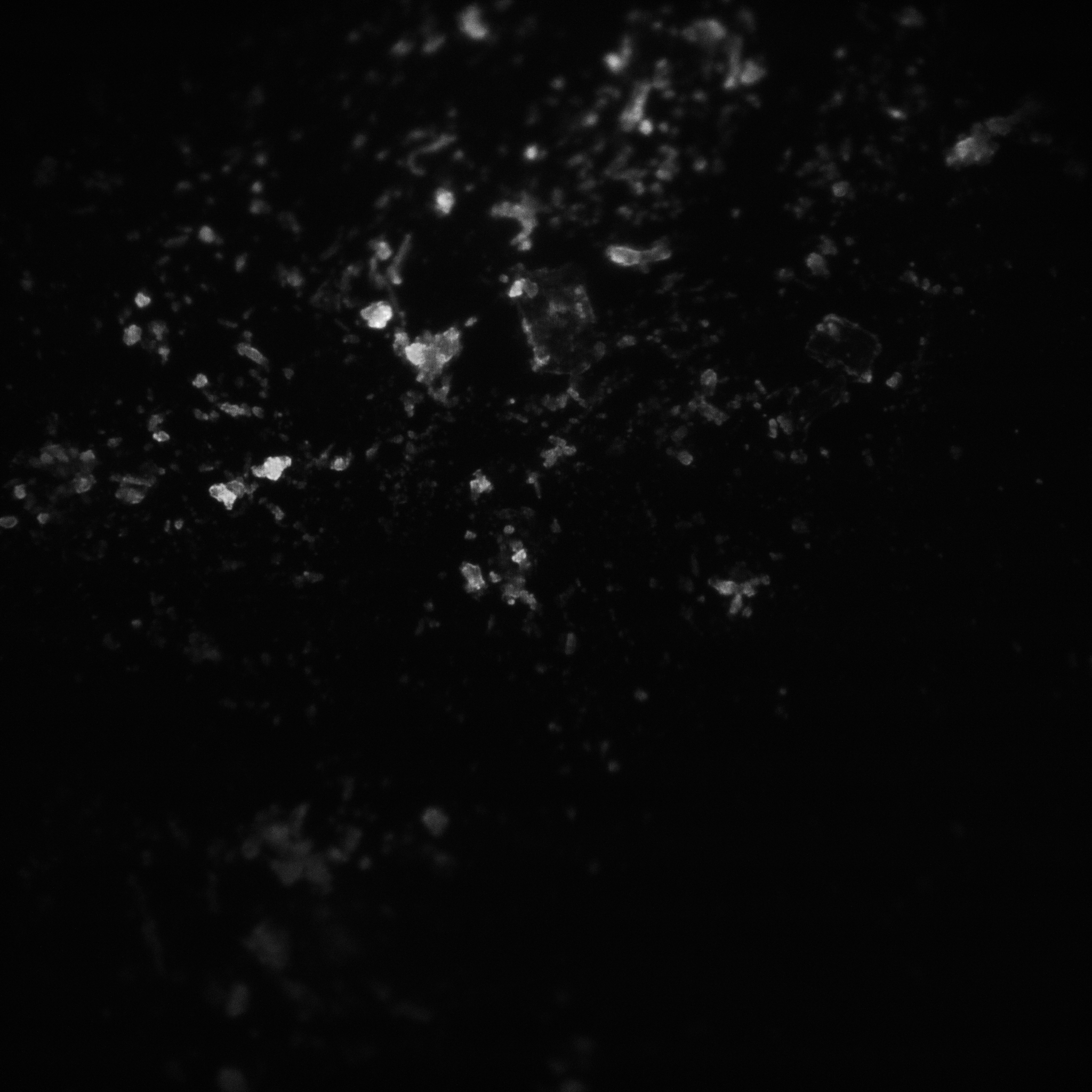

Supplement: S1 Raw Data — (ZIP) [file pone.0221254.s001.zip › Supporting_Information/fig11_motor_step/1_360.png]

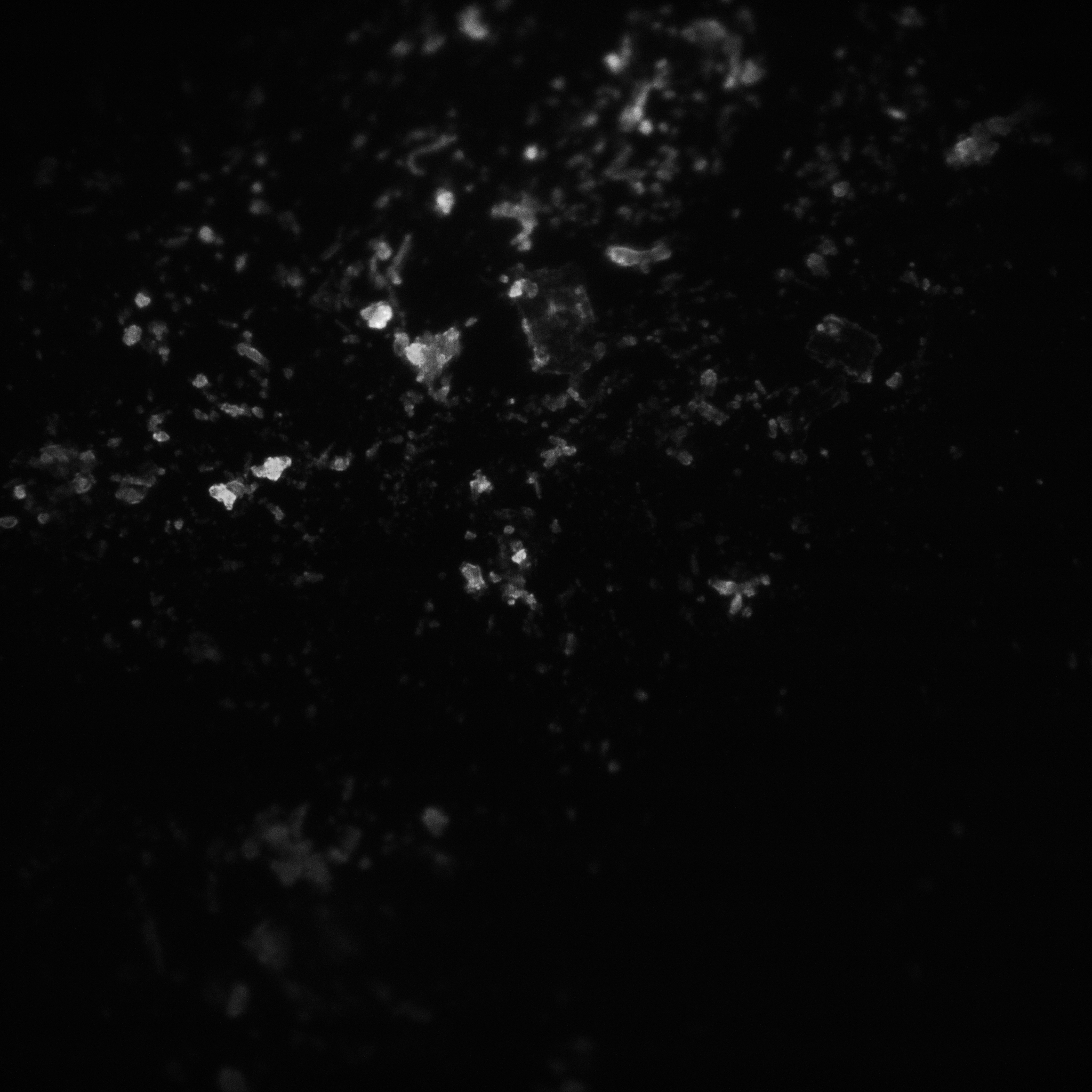

Supplement: S1 Raw Data — (ZIP) [file pone.0221254.s001.zip › Supporting_Information/fig11_motor_step/2_330.png]

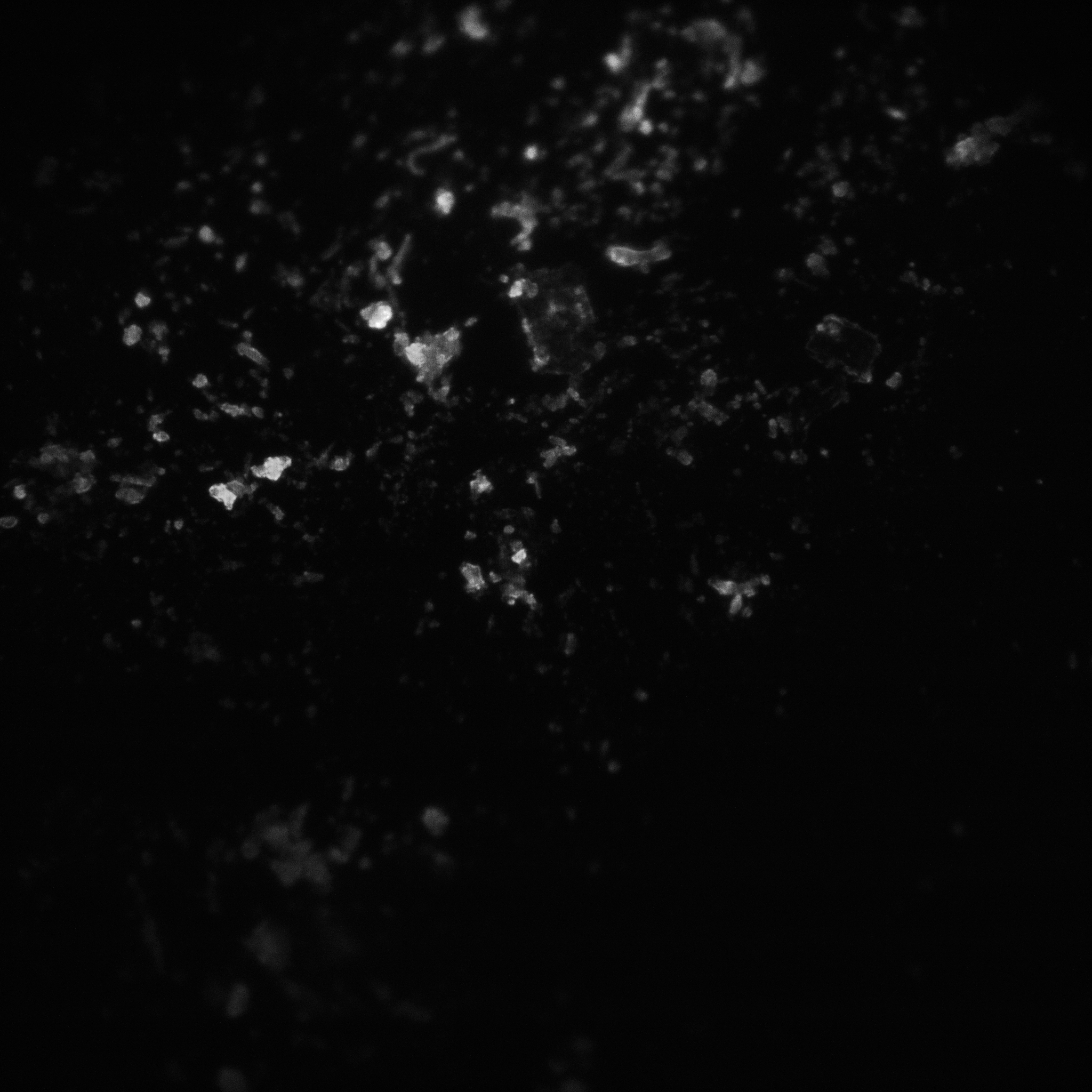

Supplement: S1 Raw Data — (ZIP) [file pone.0221254.s001.zip › Supporting_Information/fig11_motor_step/2_340.png]

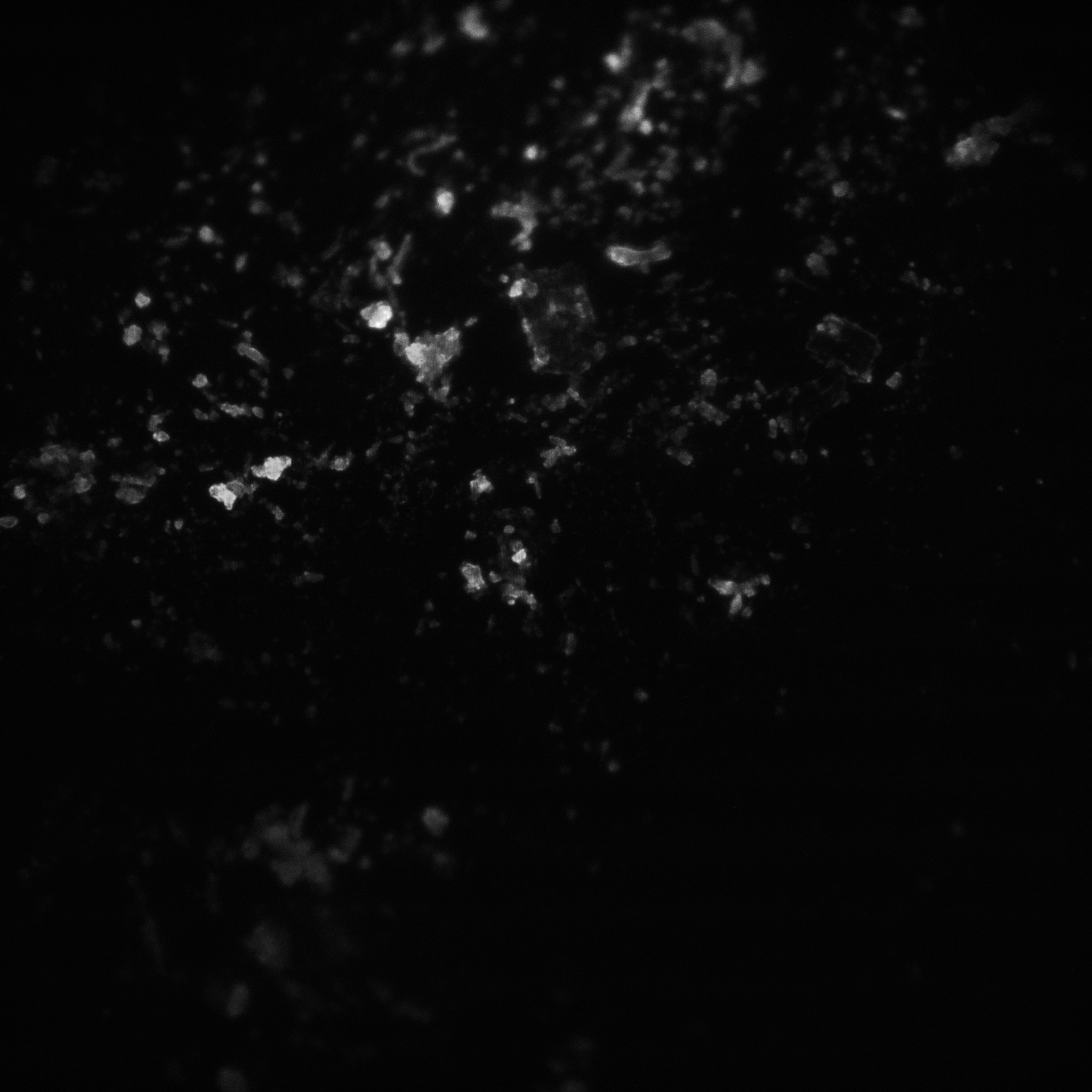

Supplement: S1 Raw Data — (ZIP) [file pone.0221254.s001.zip › Supporting_Information/fig11_motor_step/2_350.png]

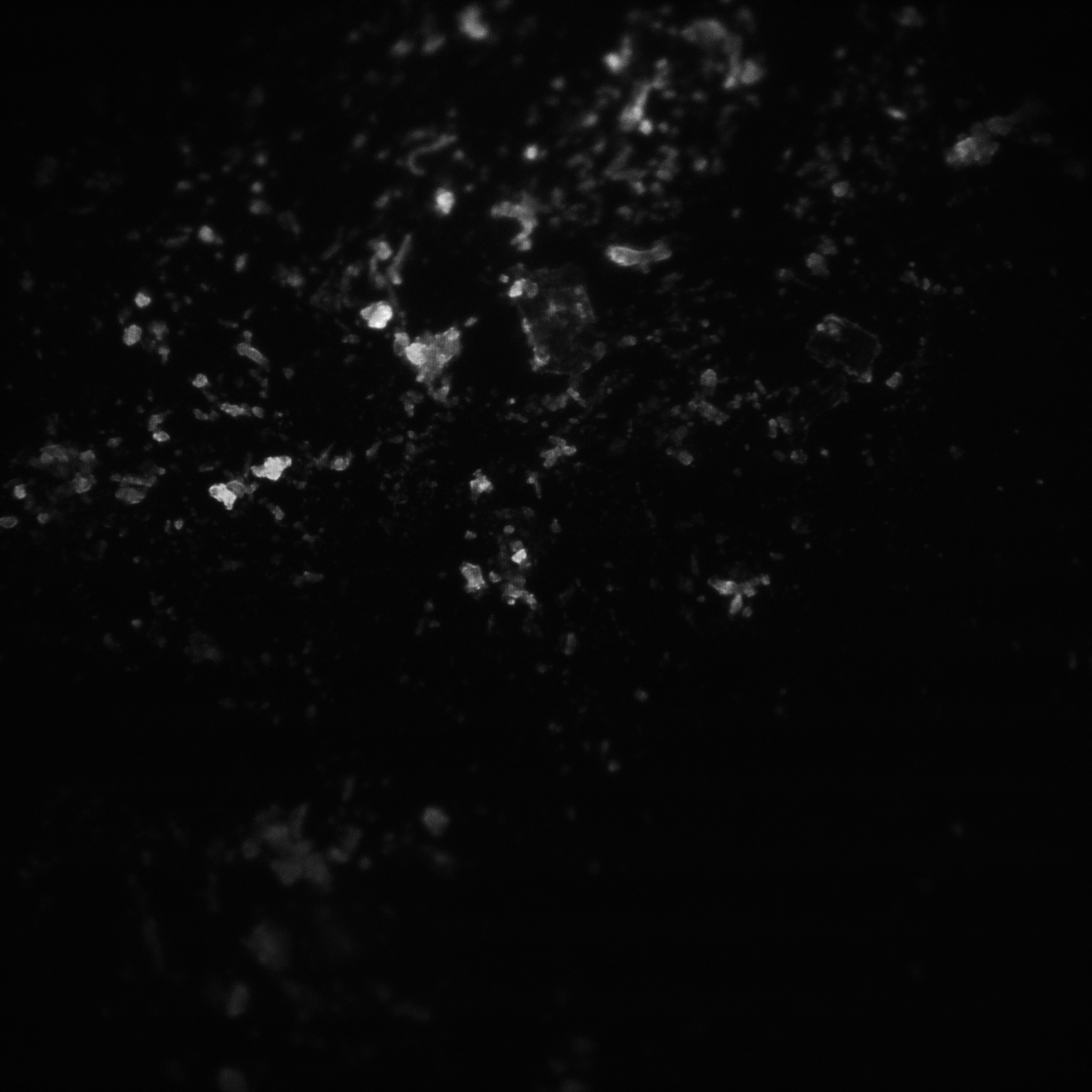

Supplement: S1 Raw Data — (ZIP) [file pone.0221254.s001.zip › Supporting_Information/fig11_motor_step/2_360.png]

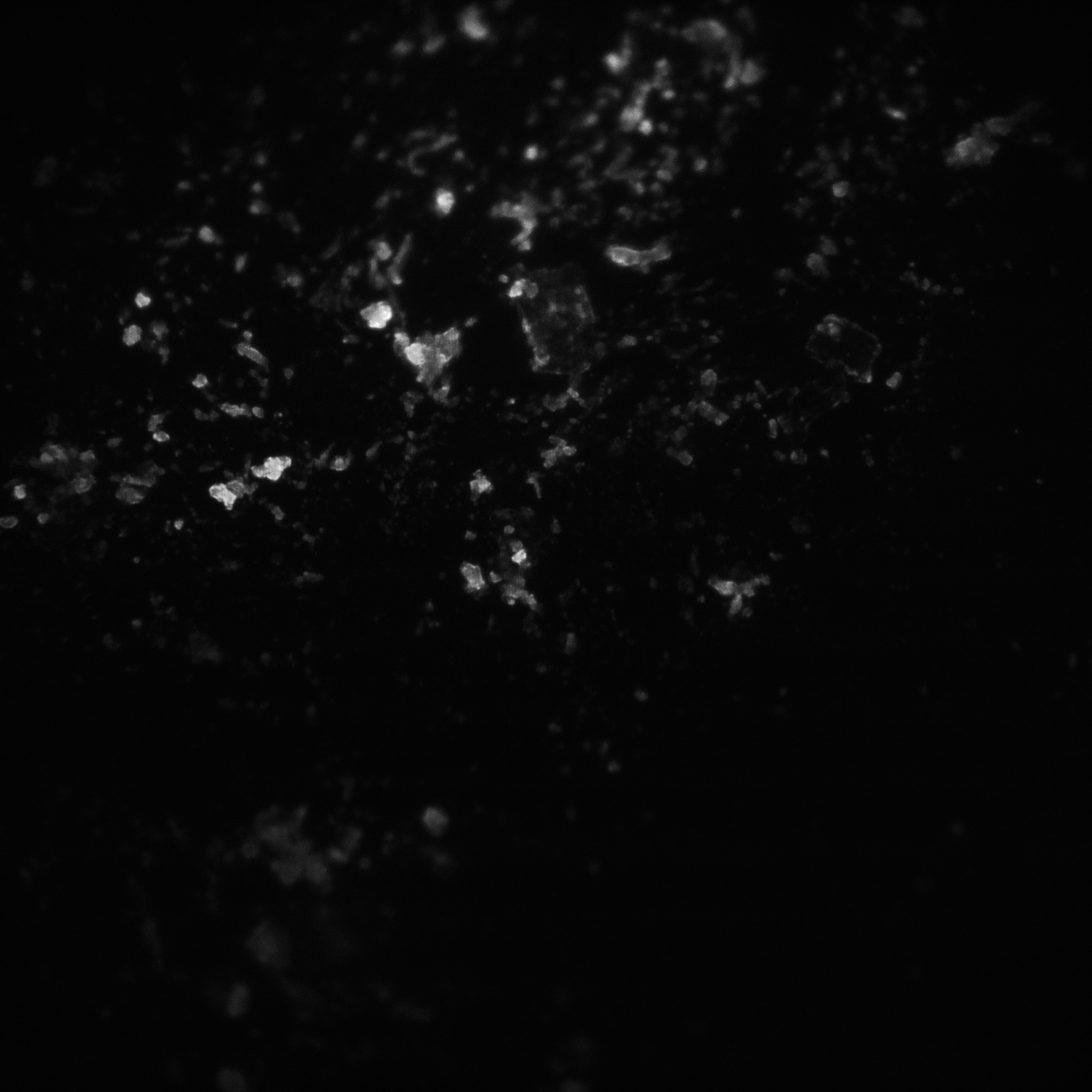

Supplement: S1 Raw Data — (ZIP) [file pone.0221254.s001.zip › Supporting_Information/fig11_motor_step/3_330.png]

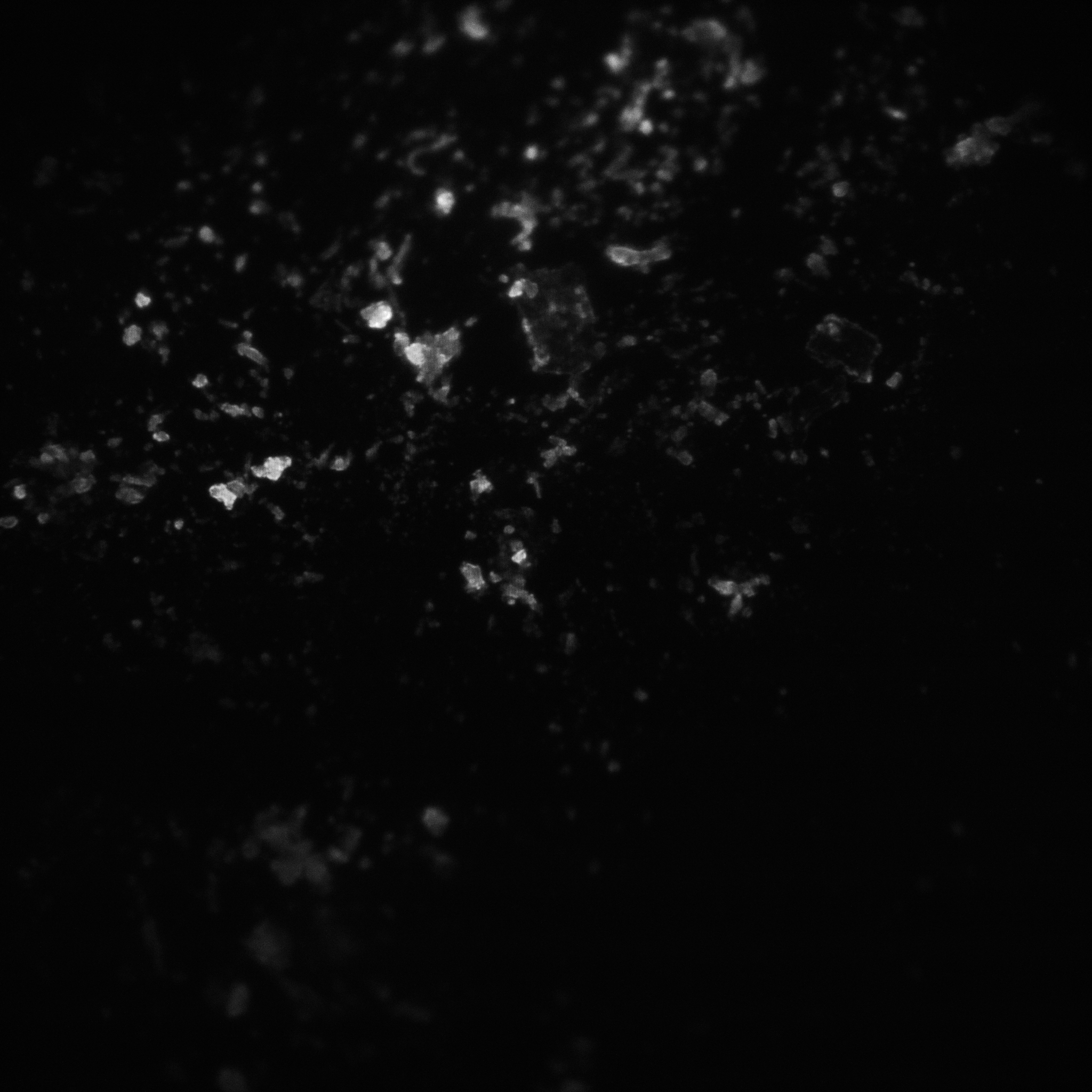

Supplement: S1 Raw Data — (ZIP) [file pone.0221254.s001.zip › Supporting_Information/fig11_motor_step/3_340.png]

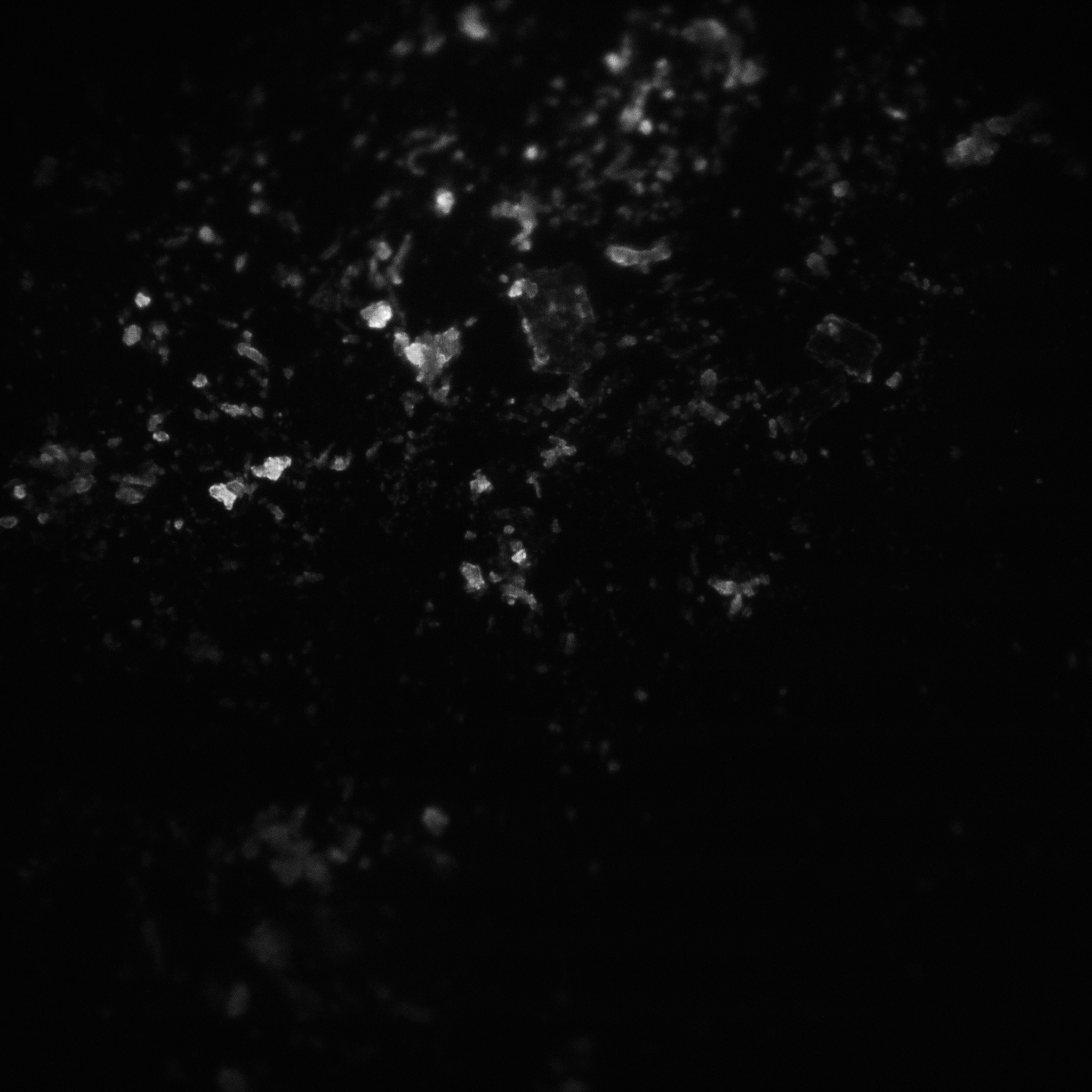

Supplement: S1 Raw Data — (ZIP) [file pone.0221254.s001.zip › Supporting_Information/fig11_motor_step/3_350.png]

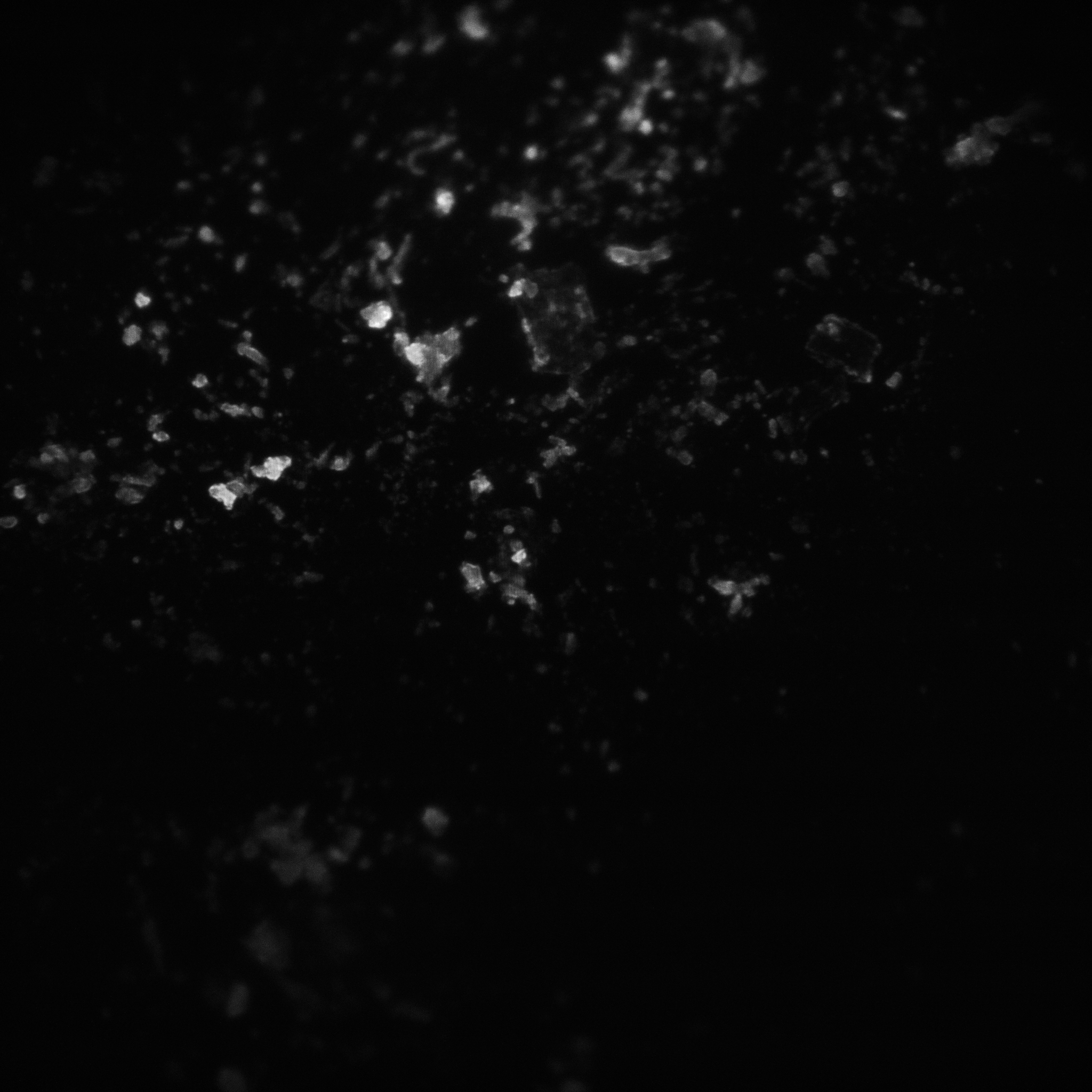

Supplement: S1 Raw Data — (ZIP) [file pone.0221254.s001.zip › Supporting_Information/fig11_motor_step/3_360.png]

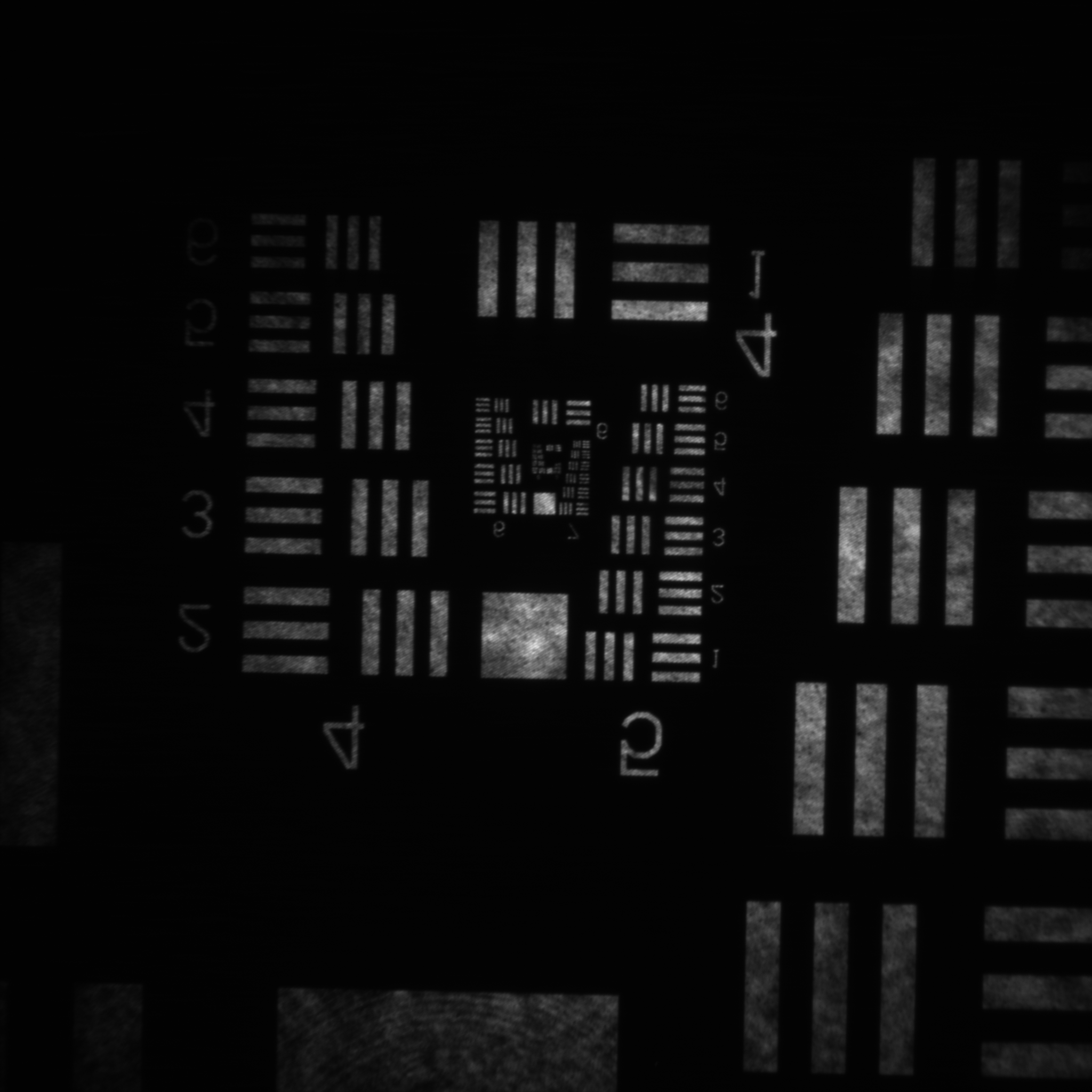

Supplement: S1 Raw Data — (ZIP) [file pone.0221254.s001.zip › Supporting_Information/Fig13USAF/01.png]

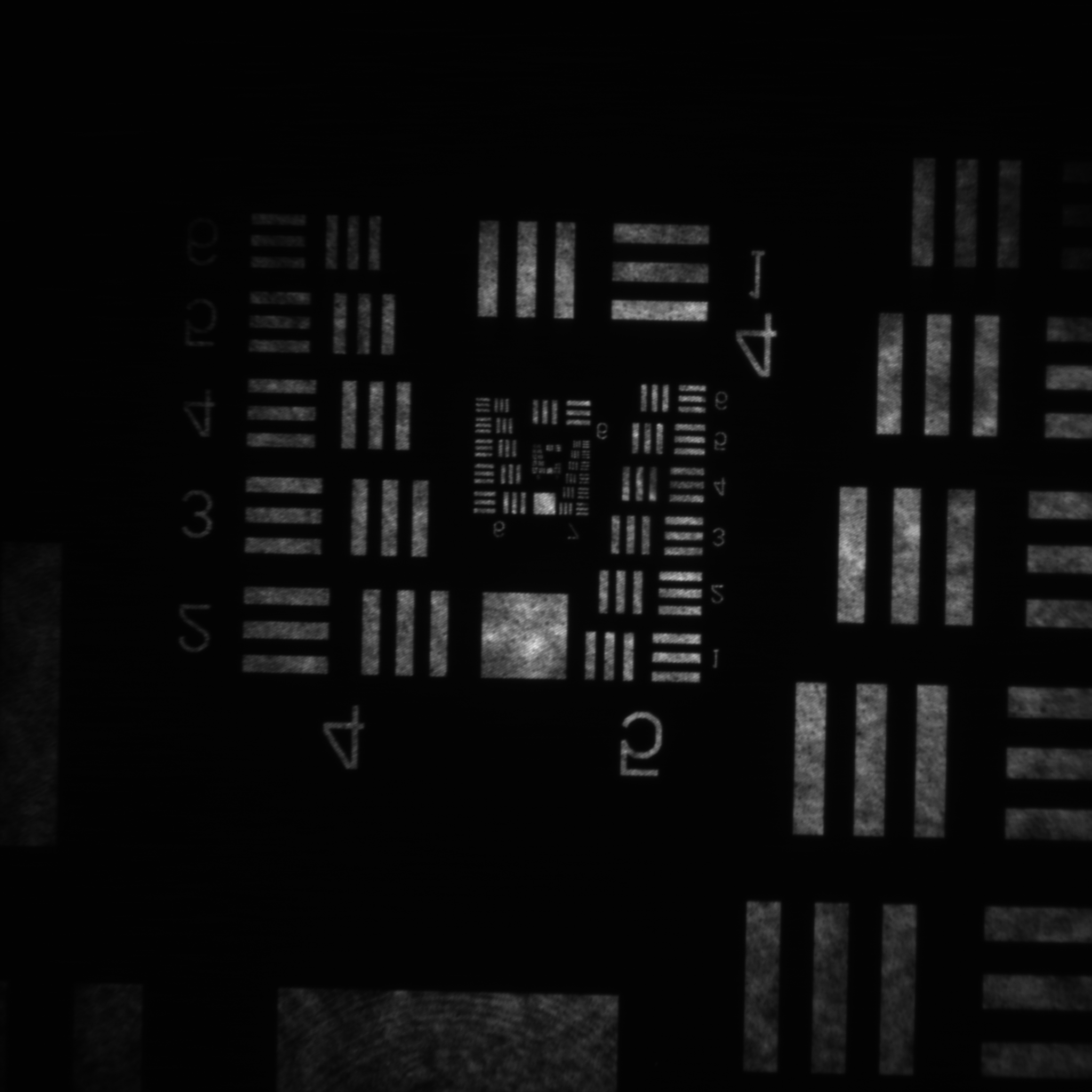

Supplement: S1 Raw Data — (ZIP) [file pone.0221254.s001.zip › Supporting_Information/Fig13USAF/02.png]

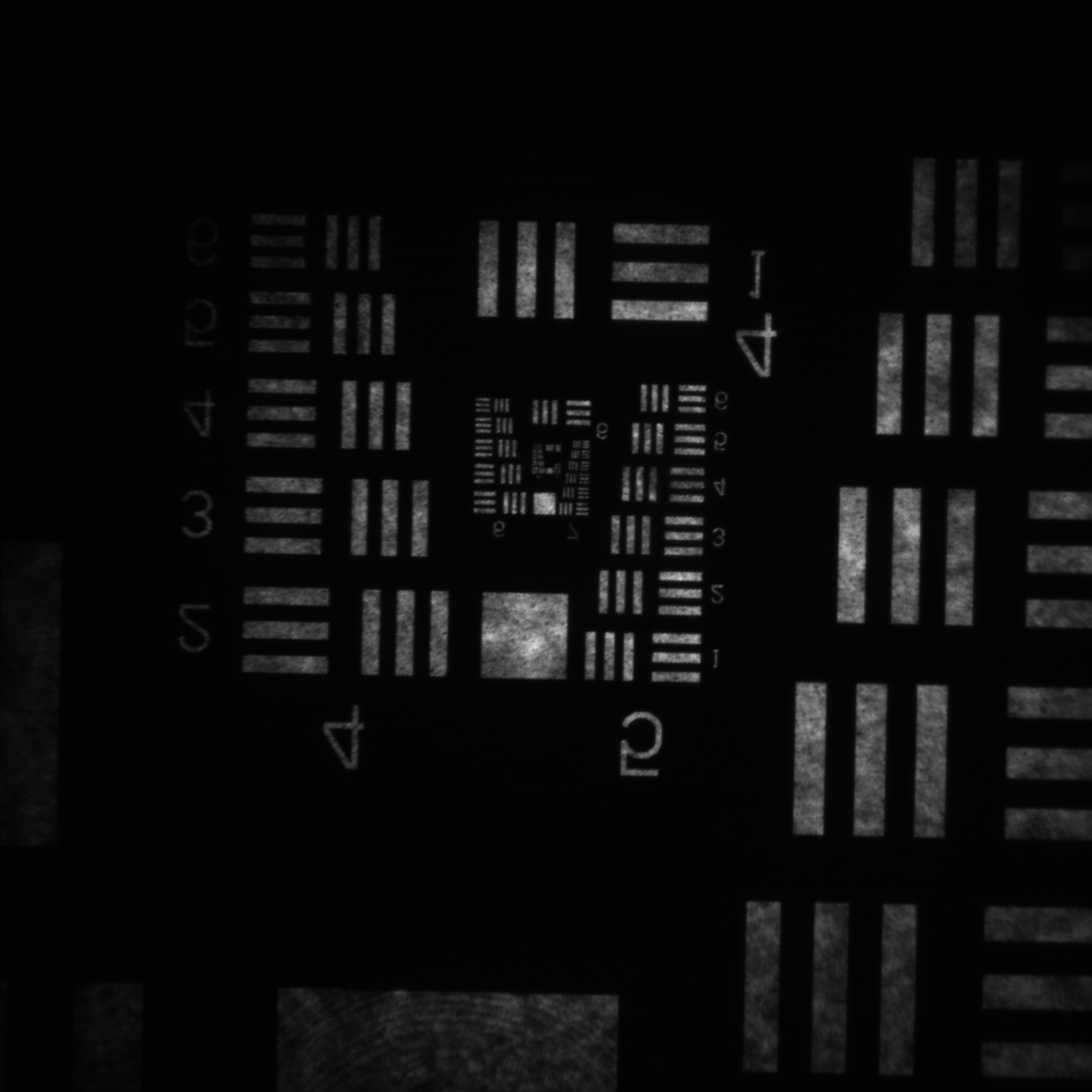

Supplement: S1 Raw Data — (ZIP) [file pone.0221254.s001.zip › Supporting_Information/Fig13USAF/03.png]

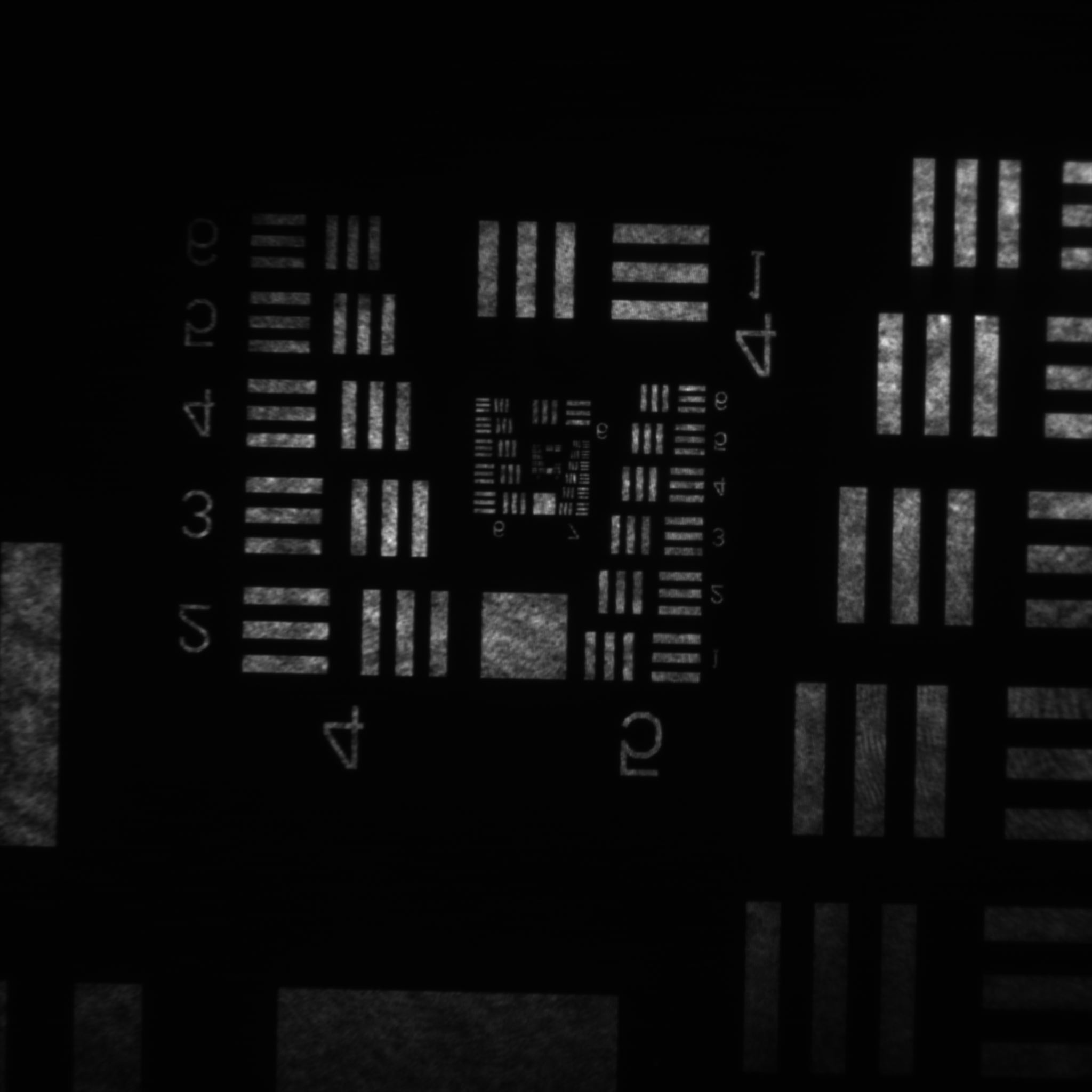

Supplement: S1 Raw Data — (ZIP) [file pone.0221254.s001.zip › Supporting_Information/Fig13USAF/M601.png]

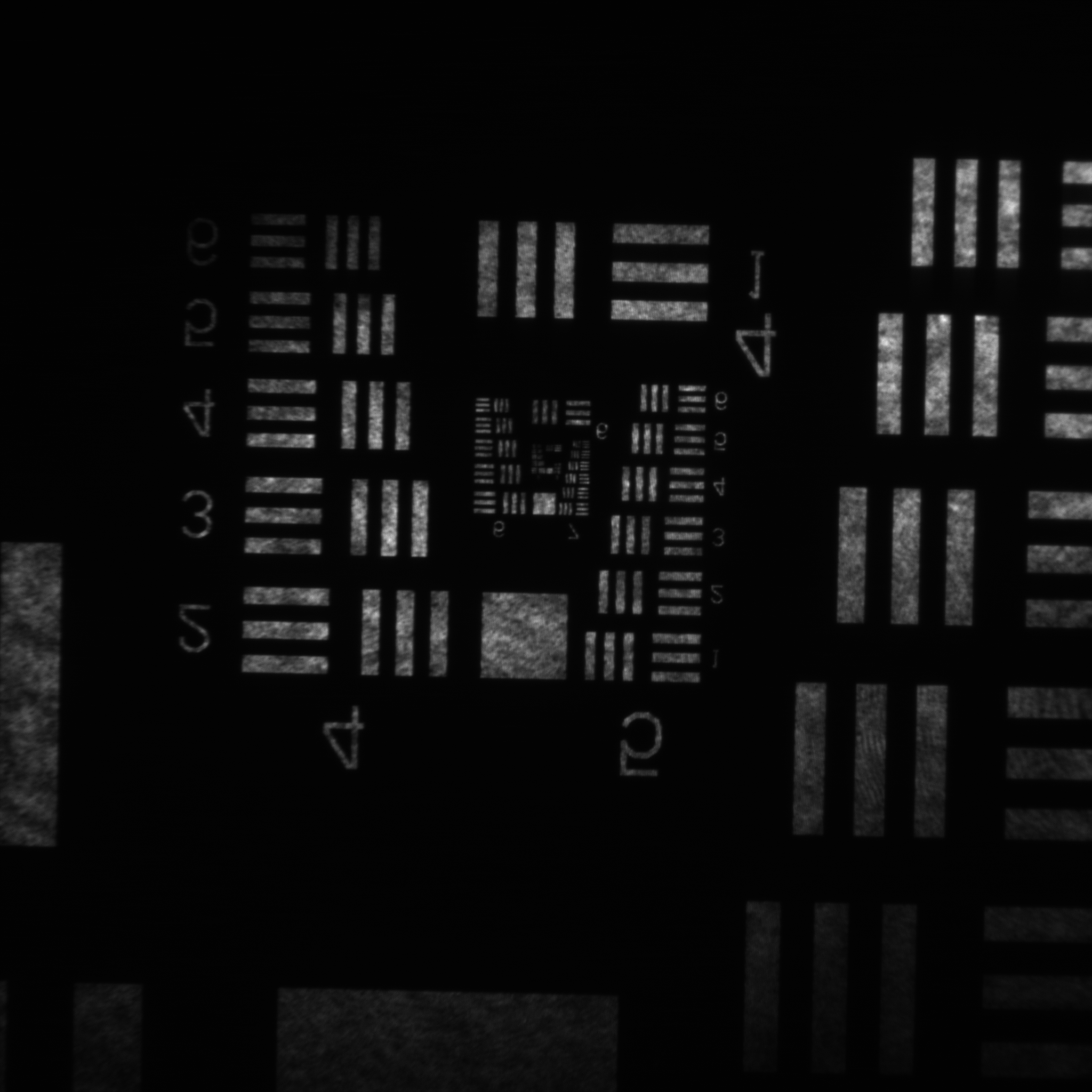

Supplement: S1 Raw Data — (ZIP) [file pone.0221254.s001.zip › Supporting_Information/Fig13USAF/M602.png]

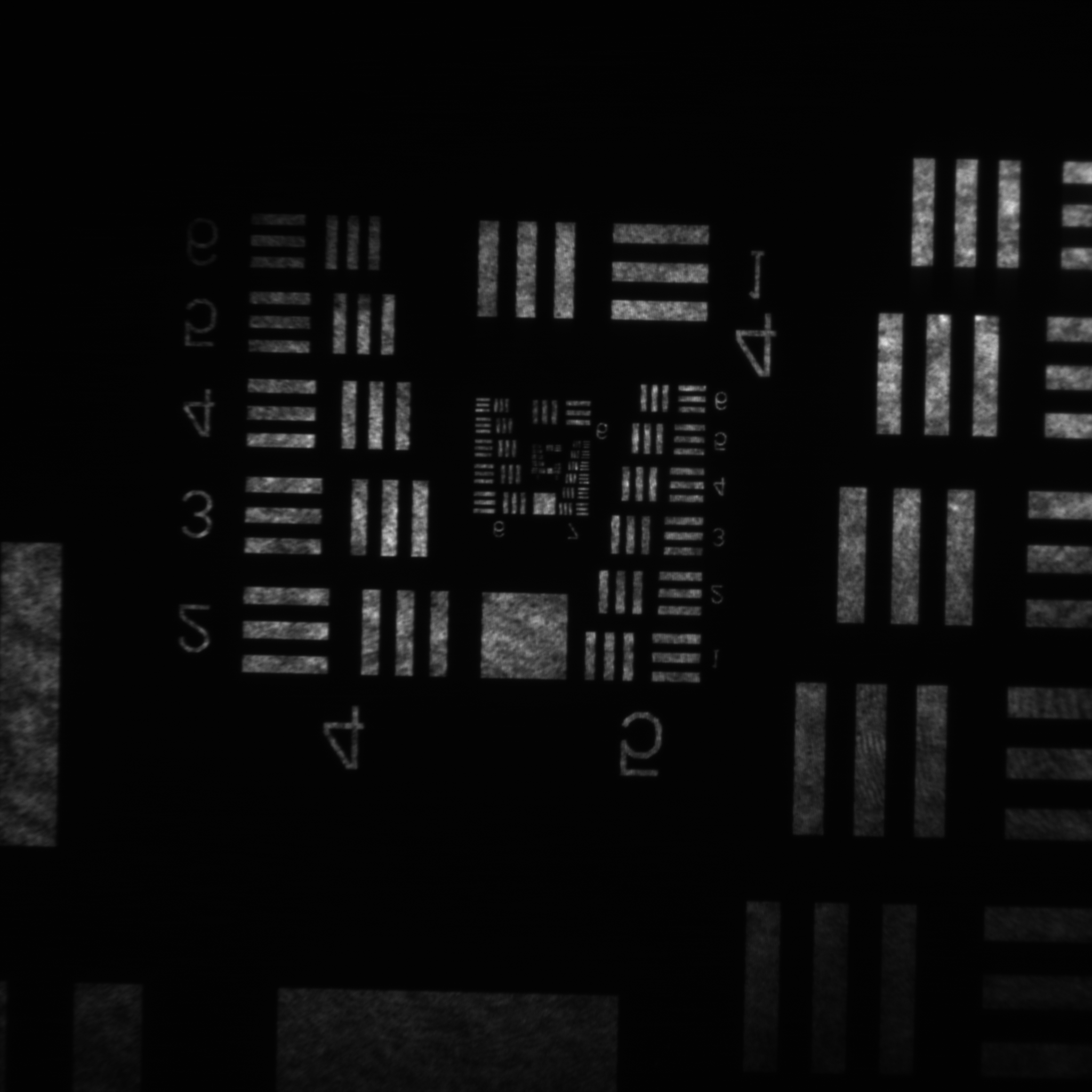

Supplement: S1 Raw Data — (ZIP) [file pone.0221254.s001.zip › Supporting_Information/Fig13USAF/M603.png]

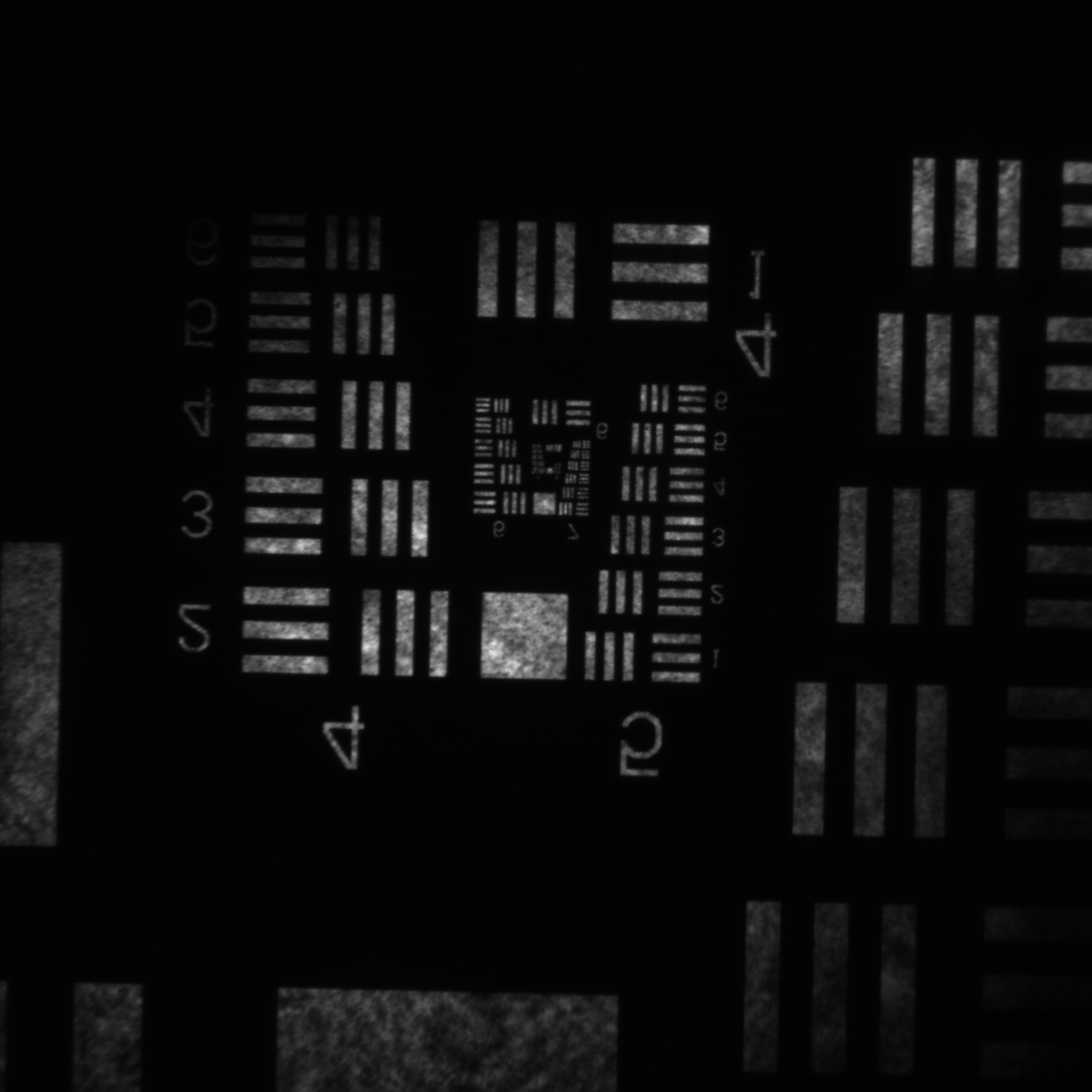

Supplement: S1 Raw Data — (ZIP) [file pone.0221254.s001.zip › Supporting_Information/Fig13USAF/P601.png]

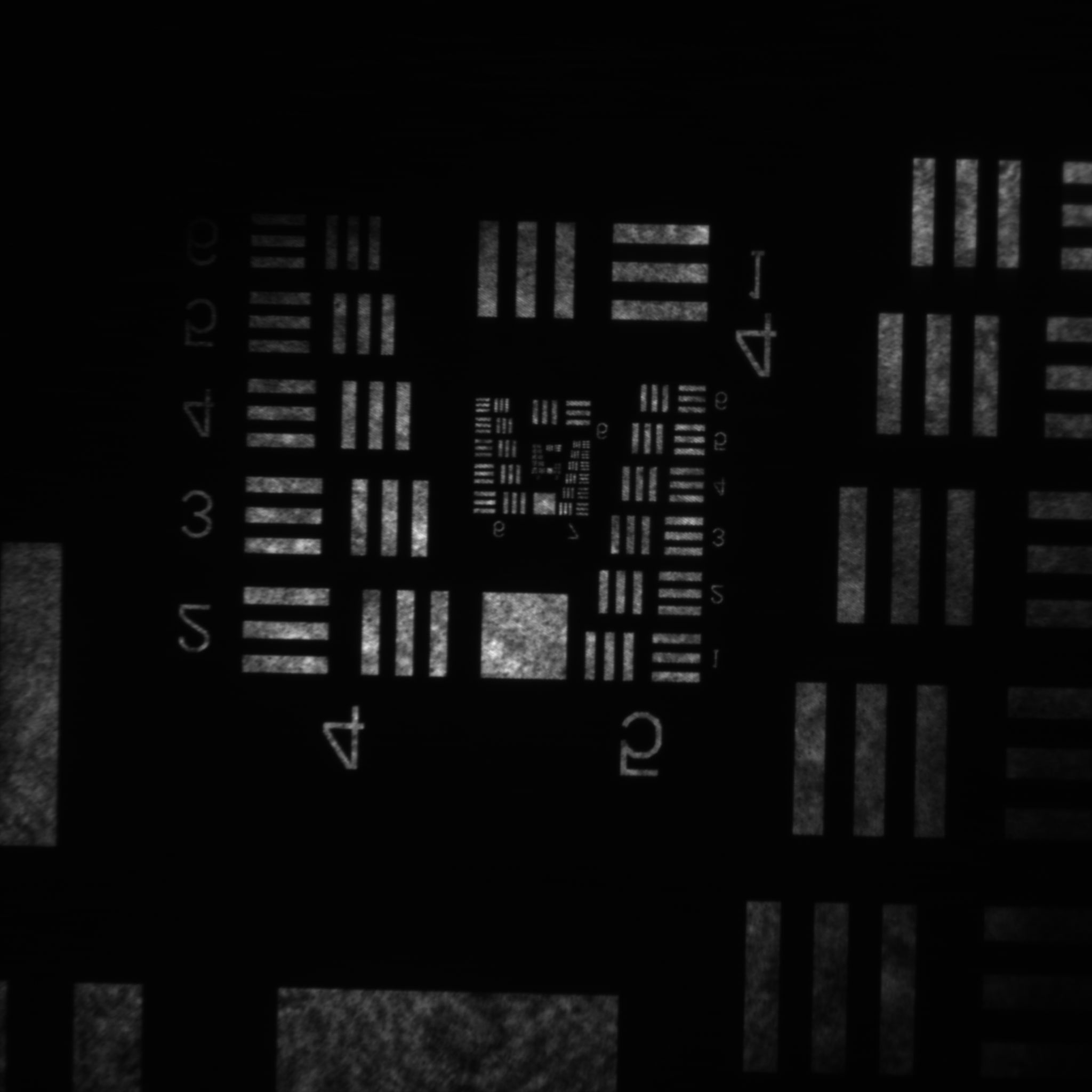

Supplement: S1 Raw Data — (ZIP) [file pone.0221254.s001.zip › Supporting_Information/Fig13USAF/P602.png]

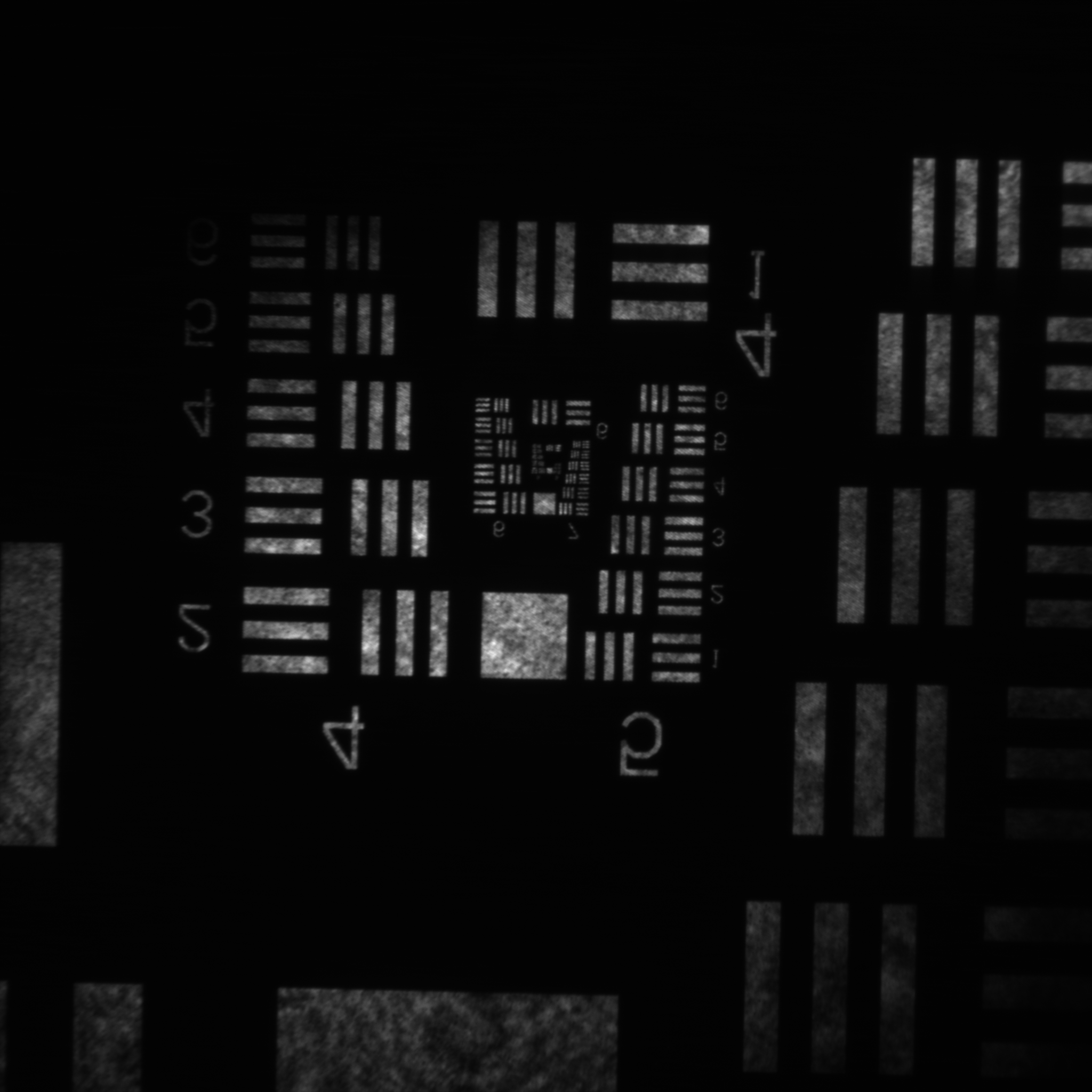

Supplement: S1 Raw Data — (ZIP) [file pone.0221254.s001.zip › Supporting_Information/Fig13USAF/P603.png]

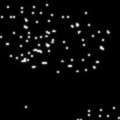

Supplement: S1 Raw Data — (ZIP) [file pone.0221254.s001.zip › Supporting_Information/Synthetic object/fig4_objectzoomedin.tif]
